# Multiple-pathway cGAS-STING activation with enhanced mild photothermal therapy through glycolysis regulation for boosting gastric cancer immunotherapy

**DOI:** 10.1016/j.mtbio.2026.102790

**Published:** 2026-01-09

**Authors:** Henan Xu, Yuxin Jiang, Ruohao Zhang, Daguang Wang, Jing Feng, Hongjie Zhang

**Affiliations:** a Department of Gastrocolorectal Surgery, General Surgery Center, The First Hospital of Jilin University, Changchun, 130021, PR China; b College of Pharmacy, Changchun University of Chinese Medicine, Changchun, 130117, PR China; c State Key Laboratory of Rare Earth Resource Utilization, Changchun Institute of Applied Chemistry, Chinese Academy of Sciences, Changchun, 130022, PR China

**Keywords:** Gastric cancer, Mild photothermal therapy, Glycolysis, cGAS-STING, Immunotherapy, Nanomedicine

## Abstract

Chemotherapy and photothermal therapy (PTT) have made prominent progress in the treatment of gastric cancer. However, the poor targeting of chemotherapeutic drugs and the thermotolerance or collateral damage induced by PTT lead to suboptimal therapeutic outcomes. To address these issues, we developed a mild PTT (mPTT) nanoparticle based on mesoporous polydopamine (MPDA), loaded with oxaliplatin (OXP) and manganese dioxide (MnO_2_), and coated with tumor cell membranes to enhance the targeting capability. On one hand, this nanoparticle disrupts glycolysis by inhibiting hypoxia-inducible factor (HIF), while suppressing heat shock proteins (HSP) to mitigate tumor "thermotolerance". On the other hand, the reactive oxygen species (ROS) generated by the MnO_2_-mediated Fenton-like reaction, OXP, and mPTT also induce immunogenic cell death (ICD) to boost adaptive immunity, as well as activate the cGAS-STING pathway through tumor DNA damage to reinforce innate immunity. The activation of both adaptive and innate immune responses triggers a potent antitumor immune reaction, which, combined with chemotherapy and enhanced mPTT, significantly suppresses tumor growth, metastasis and recurrence. This strategy not only enhances the targeting of chemotherapeutic drugs but also provides new possibilities for expanding the field of immunotherapy in gastric cancer by regulating tumor metabolism and enhancing mPTT.

## Graphical abstract

**Figure:**
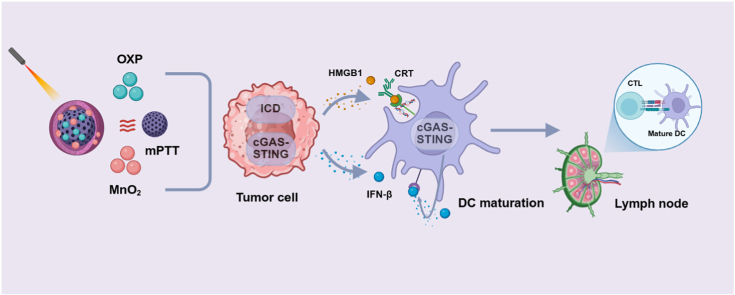

## 1 Introduction

Gastric cancer is ranked fifth globally in terms of both incidence and mortality [1]. Although its overall incidence has exhibited a downward trend over the past fifty years, gastric cancer persists as a major cause of cancer-related deaths in East Asian countries [2]. Chemotherapy is one of the primary treatment methods for gastric cancer [3]. Oxaliplatin (OXP), a third-generation platinum-based drug, mainly exerts its cytotoxic effects by inducing DNA damage and has therefore been applied in the treatment of various malignant tumors [4], including adjuvant and palliative chemotherapy for gastric cancer [5]. However, due to its poor targeting specificity and the continuous deepening of understanding about cancer, the conventional use of OXP no longer meets the therapeutic demands of contemporary oncology. Consequently, combining OXP with other treatments has provided new insights for the management of gastric cancer and its metastases.

Photothermal therapy (PTT) is a method that induces tumor cell necrosis through the photothermal effect, however, the local temperature during PTT often exceeds 50 °C, which not only causes unavoidable collateral damage to surrounding normal tissues but also subjects patients to unbearable burning sensations. To address these issues, researchers have proposed mild PTT (mPTT) with temperature below 45 °C [6]. mPTT can still induce denaturation of proteins and nucleic acids of tumor cells at 42–45 °C, thereby inhibiting tumor growth while mitigating the aforementioned adverse effects [7]. However, mPTT still cannot overcome the self-protection mechanism of tumor cells in response to external thermal stimulation, namely the expression of heat shock proteins (HSPs). HSP is detected at high levels in many types of tumors, including gastric cancer [8,9], and is closely associated with tumor initiation, progression, metastasis, and drug resistance [10]. Therefore, overcoming the high expression of HSPs induced during mPTT has become a major challenge in inhibiting tumor "thermotolerance," while, suppressing tumor adenosine triphosphate (ATP) production is an effective method to inhibit HSP expression [11]. ATP is the primary energy-supplying molecule in organisms, tumors depend on glycolysis to produce ATP, sustaining their metabolic reprogramming, this process facilitates tumor progression by affecting tumor proliferation, cell cycle, immune escape, invasion, and metastasis [12].

The production of hypoxia-inducible factors (HIFs), as a cellular response to hypoxic conditions, is involved in tumor glycolytic programming [13]. HIF-1α, as a key member of the HIF family, promotes glycolysis by upregulating pathways such as glucose transporters (GLUT) and hexokinase (HK) [14]. Several studies have confirmed that inhibiting tumor growth can be accomplished by affecting the HIF-1α/glycolysis axis [[15], [16], [17]]. In summary, alleviating tumor hypoxia can suppress tumor growth through two pathways: inhibiting glycolysis and reducing thermotolerance to improve mPTT efficacy.

In recent years, the vigorous development of immunotherapy has led to its widespread application in the treatment of gastric cancer [18]. Immunogenic cell death (ICD) refers to the process in which tumor cells, upon stimulation by external factors, undergo death while transforming from non-immunogenic to immunogenic, thereby mediating the antitumor immune response. This process relies on the release of damage-associated molecular patterns (DAMPs) from dying tumor cells, such as calreticulin (CRT) and high-mobility group box-1 (HMGB1). These DAMP molecules release "find me" and "eat me" signals to enhance the uptake of tumor cells and cellular debris by antigen-presenting cells (APCs), thereby activating the immune system [19]. Numerous approaches can induce ICD, including chemotherapeutic drugs, targeted anticancer agents, oncolytic viruses, and various physical treatment modalities [20]. In this study, related therapeutic methods such as OXP, mPTT, and the reactive oxygen species (ROS) generation have all been confirmed to induce ICD [[21], [22], [23]]. ROS is a series of reactive oxygen free radicals generated during metabolic processes, under conditions of oxidative stress, excessive ROS production can lead to severe biomolecular damage, including that of DNA and proteins [24]. There are various methods for inducing ROS generation, for example, Mn^2+^ could produce ROS through Fenton-like reactions [25]. In addition to inducing ICD, activating the STING pathway is also considered a promising approach for tumor immunotherapy. The cGAS-STING signaling pathway consists of the second messenger cyclic GMP-AMP synthase (cGAS) and the stimulator of interferon genes (STING). This pathway detects abnormal DNA to induce type I interferon responses, thereby triggering innate immunity [26,27]. The loading of OXP and MnO_2_, and mPTT induced ROS production could all lead to DNA damage. The cGAS-STING pathway precisely activates the immune system by recognizing these abnormal DNA fragments to achieve immunotherapy. Interestingly, Mn^2+^ can also enhance immune responses by increasing the sensitivity of DNA sensors cGAS and STING [28]. Furthermore, the utilization of nanoparticles to enhance immunotherapy through the activation of the cGAS-STING pathway has been comprehensively investigated [29,30].

Herein, we developed a multifunctional nanoparticle, abbreviated as MOMn@MB, which is based on mesoporous polydopamine (MPDA) loaded with OXP and MnO_2_, and coated with mouse forestomach carcinoma cell (MFC) membrane. On one hand, MPDA demonstrates exceptional biocompatibility and photothermal properties, as well as large pore structure for drug loading. The inclusion of OXP and the encapsulation with MFC cell membrane facilitate targeted drug delivery.

**Scheme 1:**
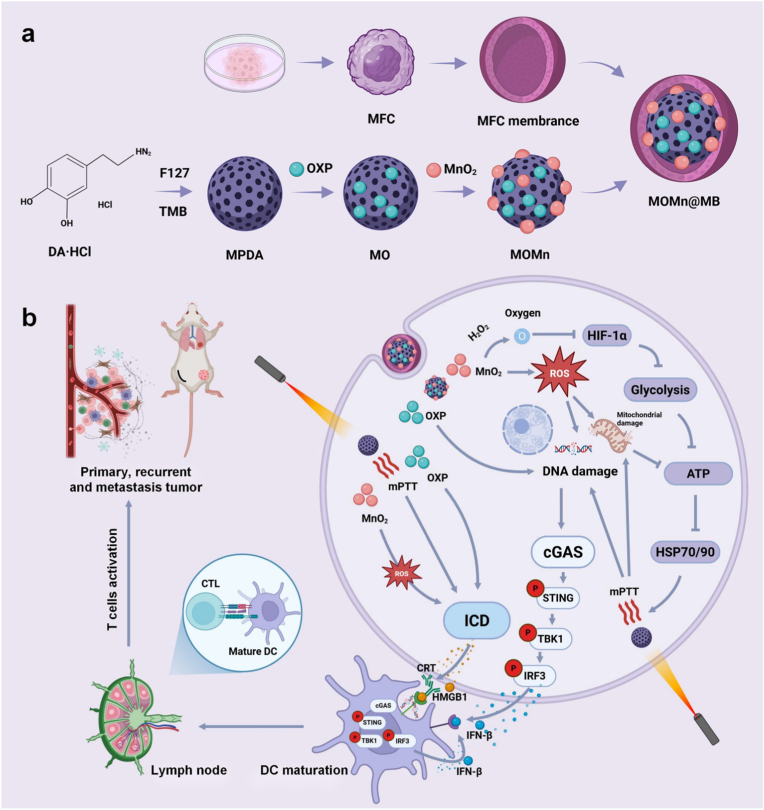
(a) Schematic diagram of the synthesis process of MOMn@MB. (b) Schematic illustration of MOMn@MB for mild photothermal-immunotherapy. Created by Biorender.

On the other hand, MnO_2_ exploits its peroxidase-like activity to decompose the excess hydrogen peroxide (H_2_O_2_) in the tumor microenvironment (TME) into oxygen [31], thereby suppressing HIF expression. This action subsequently inhibits tumor glycolysis and reduces tumor "thermotolerance" to amplify mPTT. Ultimately, MOMn@MB triggers ICD through OXP, mPTT, and ROS produced by MnO_2_ mediated Fenton-like reactions, thereby activating the immune system. Furthermore, it stimulates the cGAS-STING signaling pathway and is further sensitized by manganese ion, ultimately enhancing immunotherapy (Scheme 1). This nanoparticle achieves the synergistic integration of chemotherapy, mPTT and immunotherapy, presenting a promising therapeutic approach for gastric cancer.

## 2 Materials and methods

### 2.1 Chemicals and reagents

Potassium permanganate (KMnO_4_), dopamine hydrochloride (DA·HCl), 1,3,5-trimethylbenzene (TMB), and Triton X-100 were obtained from Shanghai Aladdin Biochemical Technology Co., Ltd. H_2_O_2_ and absolute ethanol were obtained from Sinopharm Chemical Reagent Co., Ltd. Pluronic F-127 was acquired from Sigma-Aldrich Trading Co., Ltd. Phosphate buffered saline (PBS), recombinant mouse interleukin-4 (IL-4), and recombinant mouse granulocyte-macrophage colony stimulating factor (GM-CSF) were sourced from Beijing Solarbio Science & Technology Co., Ltd. OXP was provided by Shanghai Macklin Biochemical Technology Co., Ltd. The lactic acid (LD) assay kit (colorimetric method) was obtained from Nanjing Jiancheng Bioengineering Institute. The ATP content detection kit was obtained from Beijing Boxbio Science & Technology Co., Ltd. The cell membrane protein and cytoplasmic protein extraction kit, total RNA extraction reagent (Trizol), 4′,6-diamidino-2-phenylindole (DAPI), DNA damage detection kit (γ-H2AX immunofluorescence method), Calcein-acetoxymethyl ester (AM)/propidium iodide (PI) cell viability and cytotoxicity detection kit, Hoechst live cell staining solution, ROS detection kit 2′,7′-dichlorodihydrofluorescein diacetate (DCFH-DA), and enhanced mitochondrial membrane potential detection kit (JC-1) were supplied by Beyotime Biotechnology (Shanghai) Co., Ltd. The mouse HMGB1 enzyme-linked immunosorbent assay (ELISA) kit and mouse interferon-β (IFN-β) ELISA kit were purchased from Wuhan Elabscience Biotechnology Co., Ltd. The mouse cGAMP ELISA kit was obtained from Shanghai Sinobestbio Co., Ltd. The mouse tumor necrosis factor-α (TNF-α) pre-coated ELISA kit and mouse interferon-γ (IFN-γ) pre-coated ELISA kit were obtained from Shenzhen Dakewe Biotech Co., Ltd. The calreticulin recombinant rabbit monoclonal antibody, fluorescein isothiocyanate (FITC)-labeled goat anti-rabbit antibody, cell counting kit-8 (CCK-8), anti-cGAS antibody, anti-beta-actin antibody, phosphorylated TBK1 antibody, and phosphorylated IRF3 antibody were provided by Beijing Biosynthesis Biotechnology Co., Ltd. The Annexin V-FITC/PI apoptosis detection kit was purchased from Dalian Meilun Biotechnology Co., Ltd. The anti-phosphorylated STING antibody was acquired from Cell Signaling Technology. All flow cytometry detection antibodies (APC Anti-Mouse CD3, FITC anti-mouse CD8a, FITC anti-mouse CD80, PE anti-mouse CD44, PE anti-mouse CD86, PE anti-mouse CD4, APC anti-mouse CD11c, PerCP/Cyanine 5.5 anti-mouse CD62L) were supplied by BioLegend. All reagents were used without any pretreatment prior to use.

### 2.2 Cell lines and animals

MFC and mouse fibroblast cells (L929) were cultured in Roswell Park Memorial Institute (RPMI)-1640 medium supplemented with 10% fetal bovine serum (FBS), 100 units per milliliter of penicillin and streptomycin. The culture conditions were maintained at 37 °C, with 5% CO_2_ and 95% humidity in cell incubator. For the studies in this experiment *in vivo*, 615 mice were obtained from the Institute of Hematology, Chinese Academy of Medical Sciences. This animal experimental protocol has been reviewed and approved by the Animal Ethics Committee of the First Hospital of Jilin University (Grant number: SYXK 2024-0022). All the animal experiments were strictly conducted in compliance with the guidelines for the Ethical Review of Laboratory Animal Welfare.

### 2.3 Synthesis and characterizations of MOMn@MB

#### 2.3.1 Synthesis of MPDA

DA·HCl (900 mg) and F127 (600 mg) were co dissolved in a mixed solution of ultrapure water (36 mL) and ethanol (24 mL). Then, the solution was stirred to clear and transparent. Subsequently, TMB (1 mL) was added to the above solution and stirred for 30 min. While stirring, ammonia (2.5 mL) was added quickly. After reaction for 4 h, the mixture was centrifuged at 10,000–12,000 rpm, and the black precipitate was washed several times with ultrapure water and ethanol to obtain MPDA. Finally, MPDA was dispersed in water.

#### 2.3.2 Synthesis of MO and MOM

MPDA and OXP were weighed at ratio of 1:2, dissolved in ultrapure water, and stirred for 24 h. The mixed solution was then centrifuged and washed with ultrapure water three times, resulting in MO after centrifugation, which was subsequently dispersed in ultrapure water. Loading capacity (LC) = W_*Agent*_ / W_*Total*_ × 100%. KMnO_4_ was prepared as a 10 mg/mL solution and mixed with MO at ratio of 10:1 under continuous stirring for 10 min. The mixture was centrifuged and washed with ultrapure water three times, yielding MOMn after centrifugation, which was then dispersed in ultrapure water.

### 2.3.3 Extraction of MFC cell membranes and synthesis of MPDA@MB, MO@MB, MOMn@MB

MFC cells were cultured in RPMI 1640 complete medium with FBS (10%) and penicillin-streptomycin. Cells were harvested using a cell scraper, and approximately 5 × 10^7^ MFC cells were collected by centrifugation at 3,000 rpm for 5 min. The cell pellet was then resuspended in medium. An appropriate amount of cell extraction reagent was added to the cell pellet and placed on ice for subsequent use. The cell mixture underwent repeated freeze-thaw cycles in liquid nitrogen, followed by differential centrifugation to remove intracellular contents and isolate the cell membrane. Finally, an appropriate amount of cell membrane was combined with the aforementioned different nanoparticles and extruded through a polycarbonate membrane, resulting in the synthesis of MPDA@MB, MO@MB, and MOMn@MB. The cell membrane was characterized using polyacrylamide gel electrophoresis, followed by Coomassie Brilliant Blue staining to confirm successful membrane coating.

### 2.3.4 Characterizations of MOMn@MB

Images of the size and microstructure of the nanoparticles were obtained by a transmission electron microscope (TEM Tecnai G2 F20, FEI, USA). The UV–vis–NIR absorption spectra were recorded using a UV-3600i Plus spectrophotometer (Shimadzu, Japan), and the fluorescence spectra were recorded by Fluorolog 3 (HORIBA Jobin Yvon, France). mPTT were carried out under NIR irradiation (808 nm continuous-wave diode lasers, K808D02RN-8.000W, BWT, China). The ELISA detection of cytokines was observed using a microplate reader (Multiskan FC, Thermo Fisher, USA). X-ray photoelectron spectroscopy (XPS) was detected using ESCALAB 250Xi (Thermo Fisher, USA). The content of various elements within the nanoparticles and tumor was measured by inductively coupled plasma-mass spectrometry (ICP-MS) using iCAP TQ (Thermo Fisher, USA). Immune activation within Dendritic cell (DC) and tumor tissues were detected using the flow cytometry instrument FACS Canto II (BD, USA).

### 2.3.5 Photothermal effect in vitro

MPDA@MB, MO@MB, and MOMn@MB with varying concentrations (0, 40, 60, 80, 100, and 120 μg/mL) were added to a 96-well plate (100 μL/well), followed by irradiation with an 808 nm laser (1 W/cm^2^). The temperature of the solutions was recorded at different time using a thermocouple sensor. Furthermore, four on/off cycle irradiation experiments were conducted to investigate the photothermal stability of MPDA@MB and MOMn@MB.

### 2.3.6 Oxygen generation in vitro

The MOMn@MB solution (120 μg/mL) and ultrapure water were stirred at room temperature for 30 min to remove dissolved oxygen from the liquid. Under stirring condition, 200 μL of H_2_O_2_ (100 mM) was added to each sample, and the oxygen level was measured every 20 s using a dissolved oxygen analyzer for a duration of 30 min. The same procedure was followed to measure oxygen generation by MOMn@MB without H_2_O_2_.

### 2.4 Antitumor effect *in vitro*

#### 2.4.1 Cellular uptake in vitro

MOMn and MOMn@MB were labeled by FITC to form MOMn@FITC and MOMn@MB@FITC through electrostatic adsorption, respectively. Specifically, 5 mg of nanoparticles and 1 mg of FITC were mixed in PBS (10 mL), stirred overnight, and then washed with PBS to remove excess FITC. MFC cells were implanted into 12-well plates and incubated in a constant-temperature incubator (37 °C, 5% CO_2_). After 24 h, experiment groups were replaced with RPMI 1640 complete medium containing 120 μg/mL FITC-modified nanoparticles. After uptake of MOMn@FITC and MOMn@MB@FITC (4 or 6 h), the cells were stained with/without DAPI for 15 min. Finally, the cellular uptake was detected using fluorescence microscopy/flow cytometry.

#### 2.4.2 Detection of cytotoxicity

L929 cells were cultured in RPMI 1640 complete medium and incubated in a constant-temperature incubator. Initially, L929 cells were implanted in a 96-well plate at a density of 1 × 10^5^ cells/mL and cultivated 24 h for cell attachment. Then, the L929 cells were co-incubated with MPDA@MB, MO@MB and MOMn@MB at different concentrations (200, 160, 120, 80, 40, and 0 μg/mL), respectively. The viability of L929 cells was measured using the CCK-8 assay.

MFC cells were implanted into a 96-well plate and cultivated for 24 h to allow cell attachment. Subsequently, MFC cells were co-incubated with MPDA@MB, MO@MB, and MOMn@MB at different concentrations (200, 160, 120, 80, 40, and 0 μg/mL), respectively, the irradiation groups were irradiated an 808 nm laser (1 W/cm^2^) for 10 min per well. The viability of MFC cells was detected using the CCK-8 assay.

Cell cytotoxicity was further detected by Calcein-AM and PI staining. After co-incubating MFC cells with different concentrations of the nanoparticles with/without laser irradiation, the cells were stained with Calcein-AM and PI for 15 min, then washed with PBS for monitoring by fluorescence microscopy.

#### 2.4.3 Detection of JC-1 and ATP/lactate levels in vitro

MFC cells were implanted into a 96-well plate and treated with RPMI 1640 containing MO@MB and MOMn@MB (120 μg/mL). The cells of NIR Laser groups were irradiated with an 808 nm laser (1 W/cm^2^, 10 min). All wells were co-incubated with JC-1 for 20 min and detected with fluorescence microscopy. Cell culture media from different groups were collected, and ATP/lactate levels were measured using ELISA kits.

#### 2.4.4 Detection of ICD in vitro

MFC cells were implanted in glass-bottom dishes and treated with RPMI 1640 containing MO@MB and MOMn@MB (120 μg/mL). The cells of NIR Laser groups were irradiated with an 808 nm laser (1 W/cm^2^, 10 min). To assess the level of HMGB1, the cell culture medium from different treatments was collected and measured with HMGB1 ELISA kit.

To evaluate the expression of exposed CRT, the MFC cells were further co-incubated with anti-calreticulin antibody. Subsequently, FITC-conjugated secondary antibody and DAPI was stained and detected by confocal laser scanning microscopy (CLSM).

#### 2.4.5 Cellular ROS detection in vitro

MFC cells were implanted into a 96-well plate and treated with RPMI 1640 containing MO@MB and MOMn@MB (120 μg/mL). The cells of NIR Laser groups were irradiated with an 808 nm laser (1 W/cm^2^, 10 min). Subsequently, DCFH-DA was added to each well and co-incubated for 20 min. Finally, the ROS generation was observed with a fluorescence microscope and flow cytometry.

#### 2.4.6 Cellular DNA damage detection in vitro

MFC cells were implanted into a 96-well plate and treated with RPMI 1640 containing MO@MB and MOMn@MB (120 μg/mL). The cells of NIR Laser groups were irradiated with an 808 nm laser (1 W/cm^2^, 10 min). Subsequently, the level of DNA damage in each group was detected using γ-H2AX immunofluorescence method.

#### 2.4.7 DC isolation and culture in vitro

Bone marrow cells were extracted from the femur and tibia of 615 mice. IL-4 and GM-CSF, both at a concentration of 20 ng/mL, were added with RPMI 1640, in order to purified mouse BMDCs.

#### 2.4.8 Detection of cGAS-STING pathway activity in vitro

MFC and DC cells were cultured, and treated with different nanoparticles. Subsequently, the culture medium was collected and IFN-β level was measured using an cGAMP and IFN-β ELISA kit. MFC and DC cells were harvested for Western Blot assay.

#### 2.4.9 Transcriptome analysis

MFC cells were implanted in 6-well plates and divided into the "Control group" treated with PBS and the "MOMn@MB + NIR Laser group" (n = 3). The MFC cells were collected after different treatments, and RNA sequencing (RNA-seq) for both groups was detected by Sangon Biotech (Shanghai). The R package DESeq2 was employed to analyze differentially expressed genes (DEGs) between the Control group and the MOMn@MB + NIR Laser group. A *P* value less than 0.05 and log_2_(fold change) greater than 1 were considered to indicate significantly differential gene expression. Subsequently, pathway enrichment analysis was performed using the Kyoto Encyclopedia of Genes and Genomes (KEGG), Gene Ontology (GO), and Gene Set Enrichment Analysis (GSEA) databases.

### 2.5 Antitumor effect *in vivo*

#### 2.5.1 Biosafety and long-term toxicity *in vivo*

The blood was extracted from mice, and red blood cells (RBCs) were collected by centrifugation after washing with PBS. The positive control group (ultrapure water), negative control group (PBS), and groups with different concentrations (12.5, 25, 50, 100, 200, 400, 800 μg/mL) of MO@MB or MOMn@MB, were co-incubated with RBCs for 6 h. The supernatant was collected by centrifugation (10,000 rpm, 1 min), and the absorbance was measured at a wavelength of 540 nm. The hemolysis rate was calculated as (*A*_sample_ *- A*_negative_) / (*A*_positive_ *- A*_negative_) × 100%. For long-term toxicity analysis, mice were divided into a control group and a treatment group. The treatment group received intravenous injection of MOMn@MB (4 mg/mL, 100 μL) and both groups were raised under the same conditions. After 28 days of rearing under identical conditions, routine blood analysis and liver/kidney function indicators were assessed.

#### 2.5.2 Intratumoral concentrations of nanoparticles

A suspension of 5 × 10^6^ MFC cells in PBS was subcutaneous injected (100 μL) into the left hip of mice to construct primary tumor model. Tumors were excised at 12, 24, and 48 h and dissolved in aqua regia after intravenous injections of MOMn@MB (10 mg/kg, 100 μL). The concentration of platinum in the tumors was measured using ICP-MS measurement to detect the accumulation at different time.

#### 2.5.3 Infrared thermal imaging *in vivo*

Two groups of MFC tumor-bearing mice were respectively received intravenous injections with PBS or MOMn@MB (10 mg/kg). Subsequently, both groups were irradiated with an 808 nm laser (1 W/cm^2^) for 10 min. An infrared thermal imager was used to record temperature change during irradiation at the time of 0, 2, 4, 6, 8, and 10 min.

#### 2.5.4 Effect of primary tumor treatment *in vivo*

MFC tumor-bearing mice were randomly divided into 6 groups (n = 4), Control group (PBS), NIR Laser group, MO@MB group, MO@MB + NIR Laser group, MOMn@MB group, and MOMn@MB + NIR Laser group. All administrations were performed *via* tail vein injection (10 mg/kg, 100 μL). During the treatment period, the body weight and tumor volume of the mice were recorded every other day. On the day 14, the tumors were excised for photography and weighing after all mice were euthanized. The tumors were collected for the detection of hematoxylin eosin (H&E) staining, TdT-mediated dUTP nick end labeling (TUNEL) fluorescence staining, analysis of HIF-1α, HSP70/90, and CRT immunofluorescence staining. The tumors were also collected for the analysis of expression of protein in tumor tissues by western blotting assay. Major organs (heart, liver, spleen, lungs, and kidneys) were collected for H&E staining.

#### 2.5.5 Detection of immune cells and cytokine *in vivo*

To investigate the antitumor immune responses, the tumor-draining lymph nodes (TDLNs) and primary tumors were carefully collected. Single-cell suspensions of TDLNs and primary tumors were prepared by filtration (70 μm) and washed several times with PBS.

To assess the maturation of DCs, the TDLNs suspensions were stained with APC anti-CD11c, PE anti-CD86, and FITC anti-CD80. For the primary tumor cell suspensions, APC anti-CD3, PE anti-CD4, and FITC anti-CD8a were used to determine the infiltration level of CD8^+^ T lymphocytes. The levels of IFN-β, IFN-γ, and TNF-α in the serum were measured by ELISA kits.

#### 2.5.6 Detection of pulmonary metastatic *in vivo*

1 × 10^5^ MFC cells were collected, resuspended in PBS, and injected into the tail vein (100 μL) to establish a pulmonary metastasis model. After treatment of the primary tumor (Day 14), all the mice were euthanized, and lung.

tissues were excised for photography. The pulmonary metastasis was evaluated by analyzing the size and number of lung nodules. Lung tissues were also collected for H&E staining.

#### 2.5.7 Tumor re-challenge studies *in vivo*

MFC tumor-bearing mice were randomly divided into 6 groups: Control group (PBS), NIR Laser group, MO@MB group, MO@MB + NIR Laser group, MOMn@MB group, and MOMn@MB + NIR Laser group. All administrations were performed *via* tail vein injection (10 mg/kg, 100 μL). The primary tumors were removed by surgery after different treatments. On the day 12, these mice were re-challenged with MFC cells on the other side to construct re-challenge tumors model.

### 2.6 Statistical analysis

Statistical significance between groups was analyzed using a two-tailed Student's t-test. Multigroup analyses were calculated using one-way ANOVA with Tukey post hoc analysis. All the data were expressed as mean ± standard deviation (Mean ± SD), and a *p*-value less than 0.05 was considered statistically significant. Significant differences were defined as ∗*p* < 0.05, ∗∗*p* < 0.01, and ∗∗∗*p* < 0.001.

## 3 Results and discussion

### 3.1 Synthesis and characterizations

**Fig. 1:**
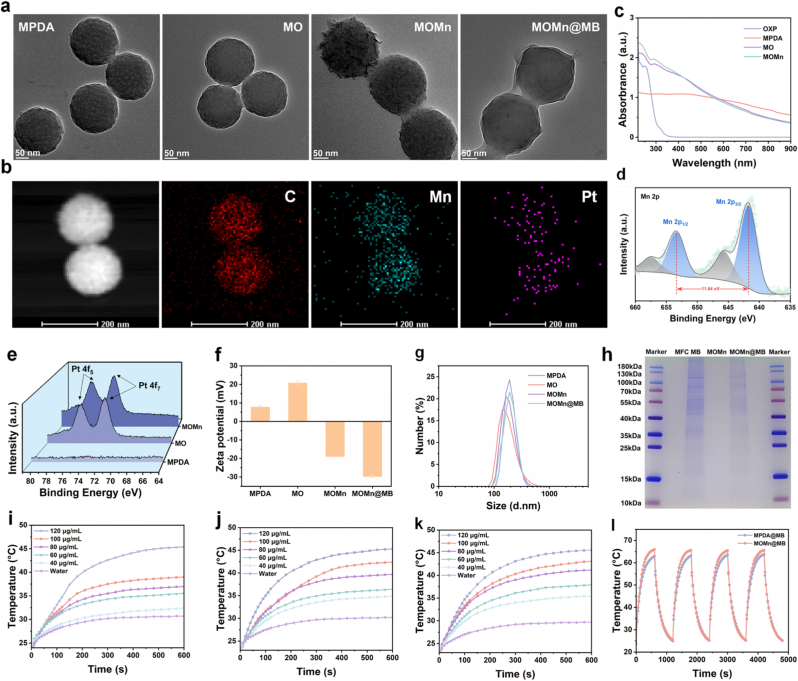
Synthesis and characterization of the MOMn@MB nanoparticles. (a) TEM images of nanoparticles. (b) Elemental mapping image of MOMn@MB. (c) UV–vis–NIR absorption spectra of different nanoparticles and OXP. XPS spectrum of (d) Mn element at the 2p orbital and (e) Pt element at the 4f orbital. (f) Zeta potentials and (g) hydrodynamic sizes of MPDA, MO, MOMn, and MOMn@MB. (h) SDS-PAGE protein analysis of MFC cell membrane, MOMn, and MOMn@MB. Samples were stained with Coomassie Brilliant Blue. Temperature change curves of (i) MPDA@MB, (j) MO@MB, and (k) MOMn@MB under laser irradiation (808 nm, 1 W/cm^2^). (l) Photothermal stability of MPDA@MB and MOMn@MB under laser irradiation (808 nm, 1 W/cm^2^). (For interpretation of the references to color in this figure legend, the reader is referred to the Web version of this article.)

MPDA was synthesized at room temperature through the self-polymerization of DA·HCl, employing F127 and TMB as templates in an alkaline environment. As depicted in the transmission electron microscopy (TEM) images (Fig. 1a), MPDA with mesoporous structure exhibits uniform spherical morphology with the size of approximately 150 nm. After loading with OXP (MO), modifying MnO_2_ (MOMn), and encapsulating with cell membrane (MOMn@MB), the morphology of MO resembles that of MPDA, whereas MOMn exhibits a rougher surface. MOMn@MB presents a membrane-like structure enveloping the nanoparticles. Elemental mappings further indicate the presence of Mn and Pt in MOMn, confirming the successful coating of MnO_2_ and loading of OXP on MPDA. (Fig. 1b).

Brunauer-Emmett-Teller (BET) specific surface area analysis reveals that MPDA has an average pore size of 4.912 nm, and the nitrogen adsorption-desorption isotherm corroborates its mesoporous structure, which is conducive to drug loading (Fig. S1). We examined the loading of OXP using ultraviolet–visible (UV–vis) absorption spectroscopy. The absorption spectrum of the OXP solution displays an absorption peak around 250 nm, while MO and MOMn exhibit analogous absorption peaks, further substantiating the successful loading of OXP (Fig. 1c). MPDA shows no significant peak due to the absence of drug loading. Moreover, ICP-MS measurements determined the OXP loading capacity (LC) to be 2.44%.

XPS revealed that the Mn 2p spectrum (Fig. 1d) exhibited two characteristic binding energy peaks at 653.35 eV and 641.71 eV, corresponding to Mn 2p_1/2_ and Mn 2p_3/2_, respectively, with an energy separation of 11.64 eV. This value is consistent with previously reported data for MnO_2_ [32,33]. XPS also confirmed the presence of carbon, nitrogen, oxygen, platinum, and manganese in MOMn (Fig. 1e and Fig. S2).

Zeta potentials of MOMn@MB decreased from −19.0 mV to −29.8 mV compared with MOMn, which is attributed to the presence of negatively charged cell membranes (Fig. 1f). The results of the hydration particle size analysis indicate that MOMn@MB, exhibits a slightly larger size compared with the other three nanoparticles due to the cell membrane coating (Fig. 1g). Subsequently, to compare the potential disparities between nanoparticles within the physiological environment and those in the TME, we measured the hydrated particle size of MOMn@MB under conditions of pH 7.4 and pH 6.5, respectively. The results showed that no significant changes with the nanoparticles were observed under different environments (Fig. S3a). Since PBS and RPMI 1640 were used as solvents for MOMn@MB in animal experiments and cell experiments, respectively, we tested the hydrated particle sizes of MOMn@MB in PBS and RPMI 1640. Finally, as H_2_O was used as the solvent for the long-term storage of MOMn@MB, we monitored dimensional changes over 7 days in H_2_O. The results indicated that the size of MOMn@MB showed no obvious change when dissolved in PBS and RPMI 1640 (Fig. S3b), while its hydrodynamic size exhibited minimal fluctuation when dissolved in H_2_O and continuously monitored for 7 days (Fig. S3c). These results collectively confirm the structural integrity and stability of MOMn@MB, providing a guarantee for the conduct of subsequent biological experiments.

To verify the successful coating of MFC cell membranes, sodium dodecyl sulfate-polyacrylamide gel electrophoresis (SDS-PAGE) was performed to analyze the proteins in MFC cell membranes, MOMn, and MOMn@MB (Fig. 1h). The results showed no apparent protein bands for MOMn due to the absence of membrane-associated proteins. In contrast, compared with MFC cell membranes, MOMn@MB exhibited similar bands at 120, 80, 55, 40, and 35 kDa, demonstrating the intact retention of MFC cell membranes on MOMn@MB.

### 3.2 Evaluation of the photothermal properties

To achieve effective mPTT effect on tumor cells, the photothermal conversion capability and the accumulation of the nanoparticles within the tumor cells are particularly crucial. Therefore, we employed MFC cell membranes to encapsulate all nanoparticles, aiming to enhance the uptake capacity of tumor cells. To investigate the photothermal performance, we recorded the temperature change of different nanoparticles (MPDA@MB, MO@MB, and MOMn@MB) at various concentrations (120, 100, 80, 60, and 40 μg/mL) under 808 nm laser irradiation (1 W/cm^2^, 10 min). The results indicated similar temperature change curves for all three nanoparticles, with a positive correlation to concentration (Fig. 1i–k). Subsequently, by plotting the temperature change curves of MPDA@MB and MOMn@MB (120 μg/mL) during four heating/cooling cycles, we observed that the peak temperature of nanoparticles did not decrease after four irradiation cycles, demonstrating excellent photothermal stability (Fig. 1l). We then calculated the photothermal conversion efficiency (η = 35%) (Table S1) of MPDA@MB and MOMn@MB according to the previous method [34]. These results suggest that MOMn@MB exhibit favorable photothermal performance and stability, making it an ideal photothermal material.

### 3.3 Evaluation of the catalase-like activity

As we have previously demonstrated the successful loading of MnO_2_, we then assessed the catalase (CAT)-like activity of MOMn@MB. Using a dissolved oxygen meter to measure oxygen production, we found that neither the aqueous solution containing H_2_O_2_ (100 mM) nor the MOMn@MB (120 μg/mL) solution without H_2_O_2_ exhibited significant oxygen generation. However, when the same concentration of H_2_O_2_ was added to the MOMn@MB (120 μg/mL) solution, the notable increase in oxygen production was detected (Fig. S4). This confirms that MOMn@MB possesses excellent CAT-like activity, capable of decomposing H_2_O_2_ in the solution into water and oxygen.

### 3.4 Cellular uptake and cytotoxicity assays

To demonstrate the targeting ability of MOMn@MB after cell membrane coating, we conducted cellular uptake experiments before evaluating the *in vitro* antitumor effect. A fluorescent probe, FITC, was adsorbed onto MOMn and MOMn@MB, then co-incubated with MFC cells. The cellular uptake was observed at different time intervals (4 h and 6 h).

**Fig. 2:**
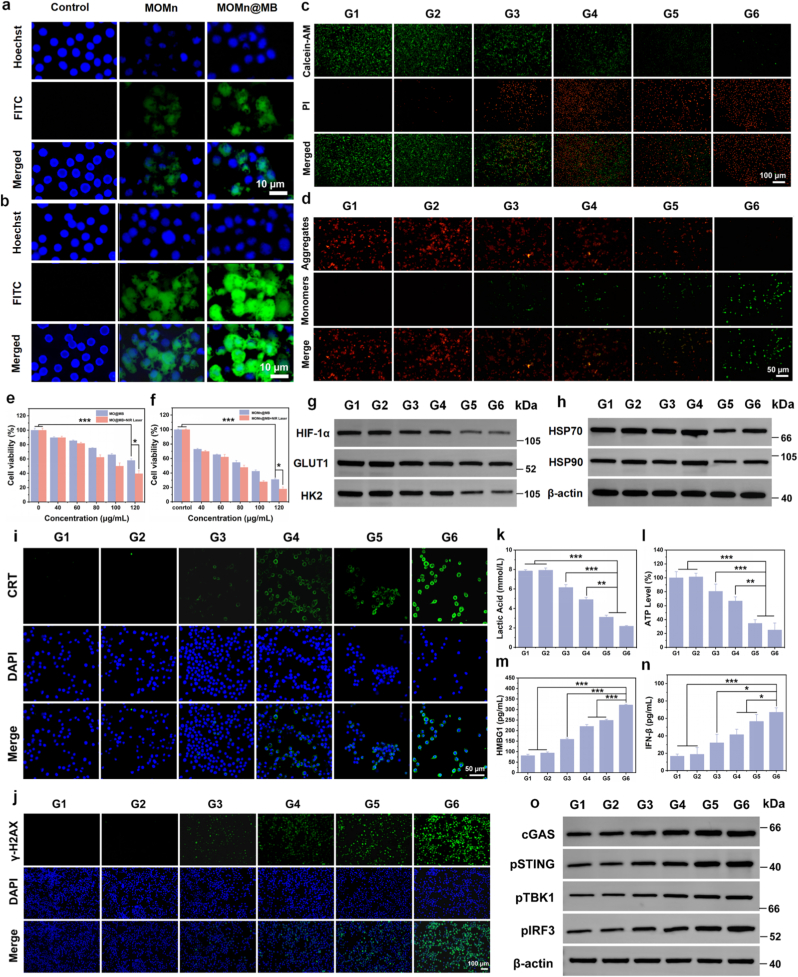
*In vitro* antitumor efficacy of the nanoparticles, ICD induction, and activation of the cGAS-STING pathway. Uptake of MOMn and MOMn@MB by MFC cells at (a) 4 and (b) 6 h. (c) Calcein-AM/PI and (d) JC-1 staining fluorescence images of MFC cells after treatment with different groups. Cytotoxicity analysis of MFC cells treated with (e) MO@MB and (f) MOMn@MB. Western blot analysis of (g) glycolysis pathway related proteins and (h) HSP70/90 in MFC cells treated with different nanoparticles. (i) CLSM images of CRT exposure on the surface of MFC cells after different treatments. (j) DNA damage assessment *via* γ-H2AX immunostaining in MFC cells treated with different nanoparticles. Levels of (k) lactate, (l) ATP, (m) HMGB1, and (n) IFN-β in MFC cells treated with different nanoparticles. (o) Western blot analysis of cGAS-STING pathway related proteins with different treatment. ∗*p* < 0.05, ∗∗*p* < 0.01, ∗∗∗*p* < 0.001. G1-G6 are denoted as Control, NIR Laser, MO@MB, MO@MB + NIR Laser, MOMn@MB, and MOMn@MB + NIR Laser groups, respectively.

Upon observation with CLSM, the FITC-modified nanoparticles exhibited green fluorescence. As depicted in Fig. 2a and b, the green fluorescence of both MOMn and MOMn@MB intensified with prolonged co-incubation time. However, within the same time frame, MOMn@MB displayed stronger green fluorescence compared with MOMn. Similarly, in the flow cytometry assay, it was also found that the intensity of intracellular green fluorescence in the MOMn@MB group was significantly increased (Fig. S5), which was highly consistent with the results observed by CLSM. This confirms that nanoparticles coated with MFC cell membranes enhance tumor cell uptake and possess superior targeting capability. Subsequently, we further evaluated the cytotoxicity of MPDA@MB, MO@MB, and MOMn@MB with various concentrations (200, 160, 120, 80, and 40 μg/mL) toward L929 cells (Fig. S6). The results indicate that when the concentration of MOMn@MB was lower than 120 μg/mL, the viability of L929 cells maintained over 80%, whereas MPDA@MB and MO@MB retained

sufficient viability even at concentrations up to 200 μg/mL. These data suggest that MPDA@MB, MO@MB, and MOMn@MB exhibit satisfactory biosafety.

Subsequently, we assessed the cytotoxic effect of MPDA@MB, MO@MB, and MOMn@MB on MFC cells using the CCK-8 assay. The results indicated that MPDA@MB did not display sufficient cytotoxicity at any concentration compared with the untreated group (Fig. S7). In contrast, MO@MB and MOM@MB exhibited cell viability rates of 57.94% and 31.01%, respectively, at a concentration of 120 μg/mL (Fig. 2e). Following 808 nm laser irradiation, these rates further declined to 39.49% and 17.62% (Fig. 2f). To observe more clearly the live/dead cell ratio in different groups, we conducted Calcein acetoxymethyl ester (AM)/propidium iodide (PI) staining (Fig. 2c), the results were consistent with those of the CCK-8 assay. Notably, the G6 group achieved the strongest MFC cell killing efficacy due to the combined effect of each component.

### 3.5 Generation of ROS in tumor cells

MnO_2_ can generate ROS through the Fenton-like reaction, leading to DNA damage and thereby inhibiting tumor growth. Consequently, we employed the ROS probe DCFH-DA to measure the intracellular ROS levels in tumor cells treated with various nanoparticles. The results of CLSM and flow cytometry assay indicated that all treatment groups produced varying degrees of ROS. The G5 and G6 groups displayed more intense fluorescence signal due to the coating of MnO_2_, suggesting that MOMn@MB possesses the capability to induce ROS due to the Fenton-like reaction (Figs. S8 and S9). The higher fluorescence intensity in Group G6 compared with Group G5 may be attributed to the oxidative stress response induced by PTT, which enhances ROS production.

### 3.6 Inhibition of glycolysis and reduction of HSP expression *in vitro*

As mentioned above, MOM@MB with CAT-like activity exhibits excellent oxygen generation ability. Therefore, we further analyzed the expression of HIF-1α protein in MFC cells under hypoxia-alleviated conditions *via* Western blot. The results revealed a significant reduction in HIF-1α protein expression in G5 and G6 groups. Furthermore, Glycolysis has long been considered the primary mode of energy production and anabolism in tumors, the protein expression levels of GLUT1 and HK2, which are key downstream proteins of the HIF-1α/glycolysis axis, also exhibited downregulation (Fig. 2g). Similarly, as metabolic products of glycolysis, lactate production was lowest in groups G5 and G6 compared with other groups (Fig. 2k). These findings indicate that MOMn@MB could decompose the excess H_2_O_2_ in the TME into oxygen due to its excellent CAT-like activity, and inhibit glycolysis in tumor cells by alleviating the hypoxia.

Mitochondria also play a crucial role in tumor energy metabolism [35]. Therefore, assessing mitochondrial damage can clarify the impact of nanoparticles on tumor cell energy metabolism. It has been reported that MnO_2_-induced ROS and photothermal therapy can cause mitochondrial damage [36,37]. Thus, we further evaluated the effect of nanoparticles on mitochondrial using JC-1. After JC-1 staining, functional intact mitochondria exhibit red fluorescence, while dysfunctional mitochondria display green fluorescence. As presented in Fig. 2d, the results show that green fluorescence signal in groups G5 and G6 is significantly stronger than that in other groups, while with the G6 group shows no red fluorescence signal at all. It demonstrates that MnO_2_-induced ROS and photothermal effect have a synergistic effect on mitochondrial damage and severely impair tumor cell energy metabolism.

As the final energy-supplying molecules in cellular energy metabolism and glycolysis, we also assessed ATP production (Fig. 2l), group G6 exhibited the lowest ATP production, at only 25%. As mentioned, ATP stimulates HSP expression by being recognized by the binding domain of HSP.

Consequently, we further examined the expression levels of HSP70/90 in various groups using Western blot (Fig. 2h). The findings indicated that the hypoxic environment in G4 group has not been alleviated, ATP production was still able to activate HSP70/90 expression and exhibited the highest levels of HSP70/90 under laser irradiation. In contrast, the G5 and G6 groups showed a significant reduction in ATP production due to the alleviation of hypoxia, leading to the downregulation of HSP70/90. Notably, the G6 group maintained low levels of HSP70/90 expression even under laser irradiation.

In summary, MOM@MB can mitigate the hypoxic environment of tumor cells through its CAT-like activity, thereby inhibiting glycolysis. Furthermore, under the dual influence of MnO_2_ and mPTT, it disrupts mitochondrial function, impacting tumor cell energy metabolism, decreasing ATP production, and ultimately downregulating HSP expression to enhance photothermal effect.

### 3.7 Induction of ICD *in vitro*

It is demonstrated that OXP, mPTT, and ROS generation can induce varying degrees of ICD, which is recognized by APCs through DAMPs to activate antitumor immunity. To confirm the strong ICD induction capability of G6 group, we first measured the release of HMGB1 in tumor cells with various treatments using ELISA kit. The results indicated that the release of HMGB1 in the G6 group is the most significant, three times that of G1 group (Fig. 2m). Subsequently, we observed CRT exposure *via* immunofluorescence staining. CLSM images (Fig. 2i) display that G6 group shows the most pronounced CRT exposure, similar to the HMGB1 release pattern, and demonstrating significant differences compared with other groups. These results indicate that although OXP, mPTT, and ROS generation can induce varying degrees of ICD, the combined treatment yields the most pronounced ICD effect. This also lays the foundation for further antitumor immunotherapy.

### 3.8 DNA damage *in vitro*

DNA damage, which leads to abnormal leakage and subsequent recognition by cGAS, is a prerequisite for the activation of the cGAS-STING pathway. We utilized the γ-H2AX assay kit for the detection of DNA damage caused by OXP, mPTT, and ROS generation. γ-H2AX serves as a marker for double-strand DNA breaks, compared with the G1 group, other treatment groups displayed varying degrees of red fluorescence signal, indicating the presence of DNA damage (Fig. 2j). Notably, the G6 group exhibited the most significant fluorescence intensity, which is attributed to the combination of OXP, mPTT, and ROS, collectively causing the most severe DNA damage.

### 3.9 Activation of the cGAS-STING pathway *in vitro*

The activation of the cGAS-STING pathway can induce a type I interferon response, thereby awakening the immune system. Therefore, we first measured IFN-β and cGAMP levels in the supernatant of tumor cells (Fig. 2n and Fig. S10). The results indicated that the G6 group exhibited the highest IFN-β and cGAMP secretion due to the most severe DNA damage, coupling with the further enhancement of cGAS and STING sensitivity through manganese ion release. To further investigate the impact of nanoparticles on the cGAS-STING pathway, we used Western blot to assess the expression of pathway-related proteins in tumor cells, including cGAS, STING, pSTING, TBK1, pTBK1, IRF3, and pIRF3 (Fig. 2o and Fig. S11). The G6 group demonstrated higher expression levels of these phosphorylated proteins than other groups, which is consistent with the above results. These studies suggest that MOMn@MB possesses superior cGAS-STING activation capacity due to the dual capability of inducing DNA damage and sensitizing the pathway.

### 3.10 Activation of APC *in vitro*

DCs, due to their ability to present antigens, play a critical role in activating adaptive immunity through their maturation status. In this work, the activation of DCs may be related to their recognition of DAMPs generated by nanoparticle-induced ICD.

**Fig. 3:**
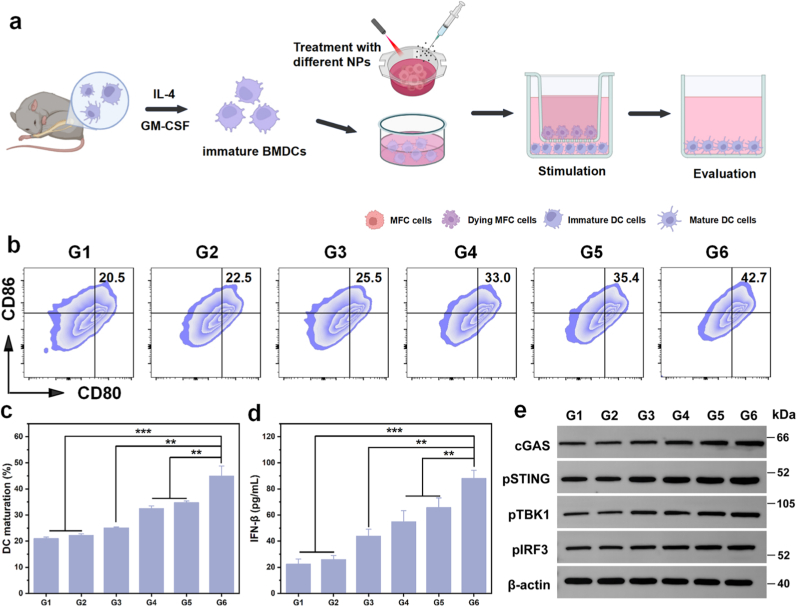
DC maturation and activation of the cGAS-STING pathway. (a) Schematic diagram of the extraction from BMDCs to DC maturation and the Transwell assay. Created by Biorender. (b) Expression of CD80 and CD86 quantitatively determined by flow cytometry analysis with different treatments, and (c) the average DC maturation rate based on the results. (d) The levels of IFN-β in DC cells with different treatments. (e) Western blot analysis was employed to evaluate the expression of cGAS-STING pathway-related proteins in DC cells across different treatments. ∗*p* < 0.05, ∗∗*p* < 0.01, ∗∗∗*p* < 0.001. G1-G6 are denoted as Control, NIR Laser, MO@MB, MO@MB + NIR Laser, MOMn@MB, and MOMn@MB + NIR Laser groups, respectively.

As depicted in the scheme (Fig. 3a), we isolated BMDCs from MFC-derived mice (615 mice) and induced them to a naive state *in vitro*. To evaluate DC maturation, MFC cells were first treated with various nanoparticles, then co-cultured with DCs, and finally analyzed using flow cytometry. Flow cytometric statistical analysis (Fig. 3b and c) indicated that the G6 group induced the highest level of DC maturation, eliciting the most pronounced ICD effect.

To further explore whether G6 also activates the cGAS-STING pathway in DCs, we utilized ELISA to detect IFN-β level in DC supernatant and Western blot to assess the expression of cGAS, STING, pSTING, TBK1, pTBK1, IRF3 and pIRF3 in DCs (Fig. 3d and e, Figure S12). As expected, the G6 group resulted in significant elevated IFN-β level and increased expression of cGAS-STING pathway-related phosphorylated proteins in DCs. These findings suggest that MOMn@MB could enhance DC maturation *via* a dual mechanism, which involves inducing ICD to release DAMPs and activating of the cGAS-STING pathway to secrete IFN-β, thereby bolstering antitumor immune activation.

### 3.11 Transcriptome analysis

**Fig. 4:**
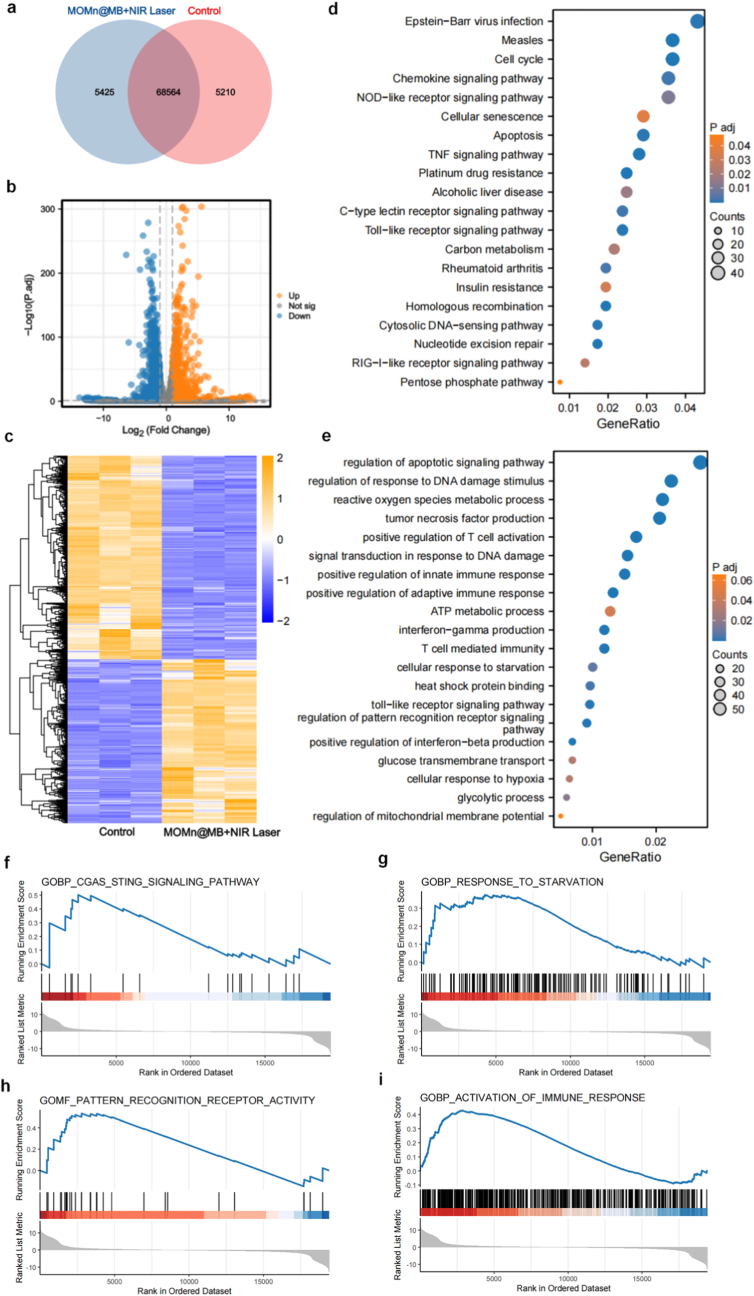
Transcriptome analysis profiles between Control and MOMn@MB + NIR laser groups. (a) Venn diagram, (b) volcano plots, and (c) heatmap of DEGs. (d) KEGG and (e) GO pathway enrichment analysis of DEGs. GSEA enrichment analysis of DEGs associated with (f) cGAS-STING signal pathway, (g) response to starvation, (h) pattern recognition receptor activity, and (i) activation of immune response in MFC cells.

To further investigate the transcriptomic changes in MFC cells after treatment, we conducted transcriptome sequencing.

analysis on cells from the Control group and the MOMn@MB + NIR Laser group. The Venn diagram (Fig. 4a) revealed 68,564 co-expressed transcripts between the two groups. Based on the volcano plot (Fig. 4b) and heatmap (Fig. 4c) with the criteria set at adjusted *P* value (*P*.adj) < 0.05 and log_2_(fold change) ≥ 1, we identified 2,730 differentially expressed genes (DEGs) in the MOMn@MB + NIR Laser group, among which 1,214 were significantly upregulated and 1,516 were significantly downregulated. The above results indicate that the MOMn@MB + NIR laser group showed significantly differentially expressed genes and unique regulatory patterns compared with the Control group.

To further clarify the specific functions and pathways affected, we conducted pathway enrichment analysis on the significant DEGs using GO and the KEGG. In the KEGG (Fig. 4d) and GO (Fig. 4e) bubble plots, we observed that the presence of MnO_2_ bestows the nanoparticles with outstanding oxygen production capabilities, resulting in a differential enhancement of glycolysis-related pathways (including carbon metabolism, glycolytic process, heat shock protein binding, etc.). Concurrently, MOMn@MB can affect tumor cell DNA *via* OXP, enhanced mPTT, and ROS, while also having the potential to induce ICD. As a result, varying degrees of enrichment were noted in pathways associated with cGAS-STING (such as the cytosolic DNA-sensing pathway, regulation of response to DNA damage stimulus, positive regulation of interferon-beta production, etc.), ICD (encompassing four receptor signaling pathways, regulation of pattern recognition receptor signaling pathway, etc.), and immune response (including positive regulation of innate immune response, positive regulation of adaptive immune response, etc.).

Finally, to more intuitively investigate the specific regulatory conditions of related pathways, we performed GSEA. As previously mentioned, the MOMn@MB + NIR laser group can significantly damage the DNA of tumor cell and possesses excellent capabilities for inducing ICD. Therefore, GSEA related to the cGAS-STING pathway (Fig. 4f and Fig. S13) showed significant upregulation, terms associated with disrupting glycolysis, like "response to starvation" (Fig. 4g), exhibited significant upregulation, while "ATP biosynthetic process" (Fig. S14) displayed significant downregulation. GSEA related to the ICD pathway (Fig. 4h) demonstrated significant upregulation. Other terms, including "activation of immune response" (Fig. 4i), "response to increased oxygen levels" (Fig. S15), "oxidative stress response" (Fig. S16), " mitochondrial depolarization" (Fig. S17), and "apoptosis" (Fig. S18), all showed significant upregulation.

These transcriptomic analysis results further corroborate the activation and regulation of relevant pathways by MOMn@MB during antitumor therapy, demonstrating the excellent capabilities of the nanoparticles in activating the cGAS-STING pathway, disrupting glycolysis, inducing ICD, and stimulating the immune system.

### 3.12 Biosafety of MOMn@MB *in vivo*

Before conducting the antitumor therapy with MOMn@MB *in vivo*, we first performed biosafety evaluation. The mice are randomly divided into two groups, one group received intravenous injections of phosphate buffered saline (PBS) solution (100 μL), while the other group was injected with MOMn@MB (10 mg/kg, 100 μL). After 28 days of rearing under identical conditions, routine blood analysis and liver/kidney function indicators of the mice were assessed (Fig. S19). The results showed no significant differences in blood parameters between the two groups, indicating the excellent biosafety of the nanoparticles. Subsequently, we conducted hemolysis analysis of MOMn@MB at varying concentrations. The results demonstrated that even at the highest concentration (800 μg/mL), the hemolysis rate in the MOMn@MB group remained below 5%, confirming the favorable biocompatibility (Fig. S20).

### 3.13 The accumulation of MOMn@MB in tumor tissues

**Fig. 5:**
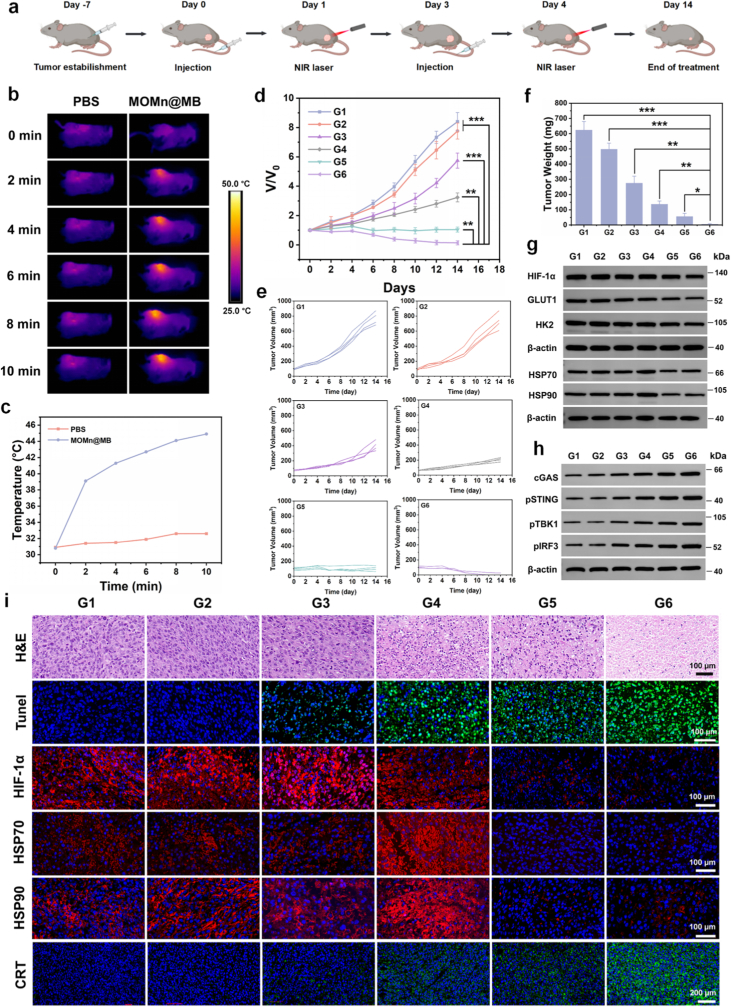
The antitumor efficacy, ICD induction, and cGAS-STING pathway activation of the nanoparticles *in vivo*. (a) Schematic diagram of the tumor-bearing mouse model establishment and treatment process. Created by Biorender. (b) Infrared thermal imaging of tumor-bearing mice and (c) temperature curves. (d) Tumor average volume change curves and (e) individual tumor volume curves with different treatments over 14 days (n = 4). (f) Average tumor weight change curves after different treatments (n = 4). (g) Western blot analysis of glycolysis-related pathways, HSP expression, and (h) cGAS-STING pathway-related proteins in tumor tissues with different treatments. (i) H&E staining images, TUNEL fluorescence staining images, HIF-1α, HSP70, HSP90, and CRT immunofluorescence staining images in tumor with different treatments. ∗*p* < 0.05, ∗∗*p* < 0.01, ∗∗∗*p* < 0.001. G1-G6 are denoted as Control, NIR Laser, MO@MB, MO@MB + NIR Laser, MOMn@MB and MOMn@MB + NIR Laser groups, respectively.

As demonstrated, MOMn@MB exhibited excellent antitumor efficacy *in vitro*. We subsequently established an MFC cell mouse model to further investigate *in vivo* antitumor effect (Fig. 5a). First of all, the accumulation of the nanoparticles in tumor tissues at different time were detected. ICP-MS revealed that the content of platinum ion in tumor tissue reached its maximum 24 h after intravenous injection of MOMn@MB and subsequently declined (Fig. S21). Therefore, in subsequent experiments, we decided to schedule all laser irradiation at 24 h post-administration.

### 3.14 Photothermal effect *in vivo*

To determine temperature change in tumor during *in vivo* treatment, we used an infrared thermal imager for real-time monitoring. The results demonstrate that 10 min after laser irradiation, the tumor temperature with the MOMn@MB + NIR Laser group rises to 44.9 °C, which precisely meets the requirement for mPTT (Fig. 5b and c), effectively inhibiting tumor growth while avoiding collateral damage to surrounding tissues.

### 3.15 Synergistic antitumor effect *in vivo*

Specifically, tumor-bearing mice were intravenously injected with PBS or different nanoparticles, with or without laser irradiation, and divided into the following groups (n = 4), Control (G1), NIR Laser (G2), MO@MB (G3), MO@MB + NIR Laser (G4), MOMn@MB (G5), and MOMn@MB + NIR Laser (G6). Compared with G1, other groups except G2 showed tumor growth inhibition, with the.

most pronounced effect observed in G6 (Figure S22, Fig. 5d–f). After tumor resection H&E and TUNEL fluorescence staining reveal that the tumor tissue in the G6 group has the most severe necrosis and apoptosis (Fig. 5i, Fig. S23). The fluorescence staining and Western blot analysis of HIF-1α and HSP70/90, as well as the protein expression of key glycolytic enzymes GLUT1 and HK2, show the lowest levels in G5 and G6 groups, which are consistent with the results *in vitro* (Fig. 5g and i, Figure S23). These results indicate that MOMn@MB could alleviate tumor hypoxia, disrupt glycolysis, and ultimately reduce HSP expression *in vivo*. Additionally, we monitored the change in mouse body weight during treatment and performed H&E staining of major organs (heart, liver, spleen, lung, and kidney). No significant differences were observed regardless of treatments (Figs. S24 and S25), further confirming the favorable biosafety of the nanoparticles *in vivo*.

### 3.16 Activation of the cGAS-STING pathway and immunotherapy *in vivo*

MOMn@MB exhibits excellent ICD-inducing capability and can activate the cGAS-STING pathway *in vitro*. Therefore, we investigated whether the nanoparticles possess the same ability *in vivo*. Immunofluorescence staining of CRT in tumor tissue sections revealed that the G6 group exhibited the strongest fluorescence intensity, suggesting the most intense exposure of CRT (Fig. 5i and Fig. S23). Western blot analysis of tumor tissues further demonstrated that this treatment group exhibited the highest expression of STING pathway-related phosphorylated proteins (Fig. 5h), whereas no significant changes were observed in the expression of non-phosphorylated proteins (Fig. S26). To further explore the mechanism of immune response triggered by ICD and the cGAS-STING pathway, we evaluated the enrichment of immune cells and the levels of cytokines *in vivo*.

**Fig. 6:**
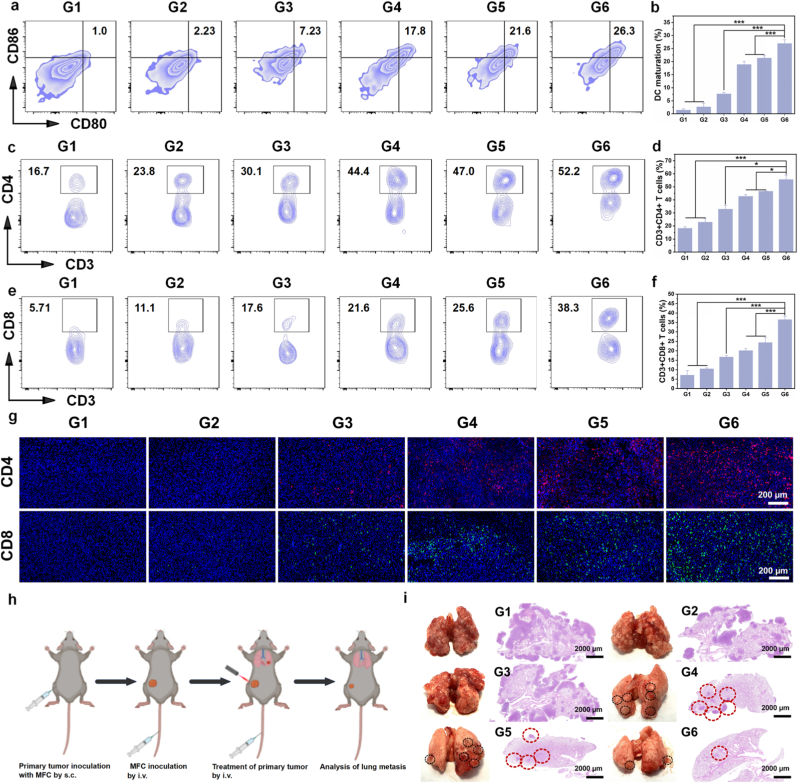
Immune response activation *in vivo*. (a) Flow cytometry analysis of DC maturation in TDLNs of mice with different treatments. (b) Statistical analysis of average DC maturation in TDLNs with various treatments. (c) CD3^+^CD4^+^ T cell infiltration in primary tumors by flow cytometry analysis with various treatments. (d) Statistical analysis of average CD3^+^CD4^+^ T cell infiltration in primary tumors with different treatments. (e) CD3^+^CD8^+^ T cell infiltration in primary tumors by flow cytometry analysis with various treatments. (f) Statistical analysis of average CD3^+^CD8^+^ T cell infiltration in primary tumors with various treatments. (g) Immunofluorescence staining of CD4^+^ and CD8^+^ cells in primary tumor tissues with various treatments. (h) Schematic diagram depicting the establishment of a lung metastasis model and corresponding treatment procedures. Created by Biorender. (i) Digital images of lung tissue and H&E staining sections following different treatments. Route of administration: subcutaneous injection (s.c.), intravenous injection (i.v.). ∗*p* < 0.05, ∗∗*p* < 0.01, ∗∗∗*p* < 0.001. G1-G6 are denoted as Control, NIR Laser, MO@MB, MO@MB + NIR Laser, MOMn@MB and MOMn@MB + NIR Laser groups, respectively.

*In vitro* experiments show that MOMn@MB promotes DC maturation through dual pathways. To validate these effects *in vivo*, TDLNs were analyzed (Fig. 6a and b). The G6 group exhibits the highest DC maturation rate, which is consistent with the *in vitro* results. As a bridge between innate and adaptive immunity, mature DCs ultimately exert antitumor immune effect by activating T cells. Therefore, we used flow cytometry to assess the proportions of helper T cells (CD4^+^ T cells) and cytotoxic T cells (CD8^+^ T cells) in tumor tissues with different treatments (Fig. 6c–f), and the G6 group exhibited the highest proportions. Additionally, immunofluorescence staining of tumor tissue sections confirmed the activation of CD4^+^ T cells and CD8^+^ T cells *in vivo* (Fig. 6g and Fig. S27). Similar to the flow cytometry results, the G6 group significantly increased the levels of CD4^+^ T cells and CD8^+^ T cells in tumor tissue sections. Given the critical role of cytokines in antitumor immunity, we also measured the levels of IFN-γ, TNF-α and IFN-β, in serum of mice using ELISA. Consistent with the flow cytometry analysis, the G6 group exhibited the highest levels of all three cytokines (Figs. S28, S29, and S30), further confirming potent ability of the nanoparticles to induce antitumor immunity.

Finally, we established a lung metastasis model (Fig. 6h) to evaluate whether the nanoparticles could inhibit distant tumor metastasis. As shown in Fig. 6i, the lung metastasis in the G3 group showed no significant difference from the G1 and G2 groups in terms of lung tissue and H&E staining, this result indicates that chemotherapy alone cannot suppress pulmonary metastasis. In contrast, due to its multi-pathway induction of antitumor immunity, the G6 group exhibited only minimal metastatic lesions in lung tissue and H&E staining, significantly suppressing pulmonary metastasis.

### 3.17 Evaluation of long-term anti-neoplastic immune memory in tumor re-challenge mode

**Fig. 7:**
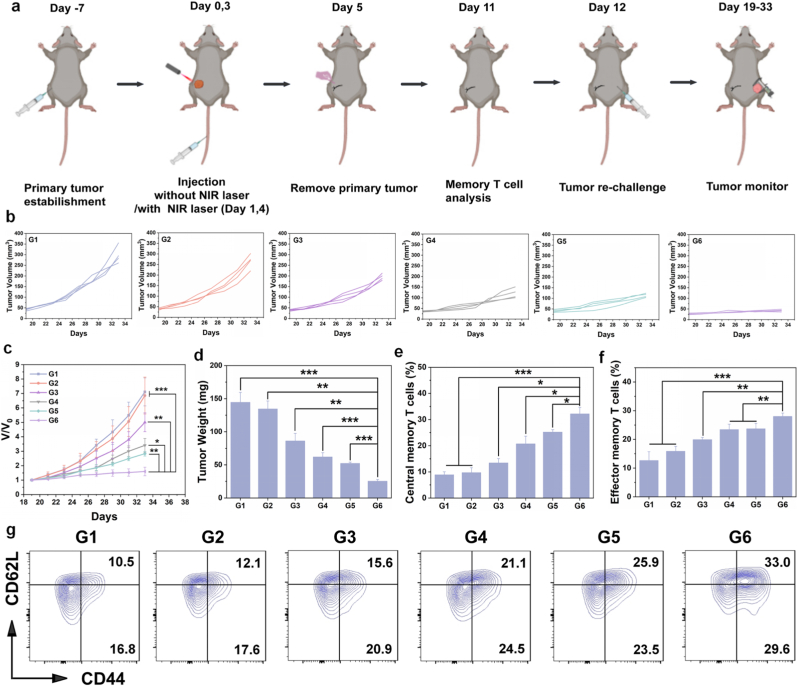
Assessment of the therapeutic efficacy and long-term antineoplastic immune memory of nanoparticles in tumor re-challenge model. (a) Schematic diagram of tumor re-challenge model establishment and treatment process. Created by Biorender. (b) Individual re-challenge tumor volume curves with different treatments (n = 4) and (c) re-challenge tumor average volume change curves (n = 4). (d) Average re-challenge tumor weight after different treatments (n = 4). Relative proportions of (e) central memory T cells and (f) effector memory T cells in spleens after different treatment. (g) Representative flow cytometry images of memory T cells in spleens. ∗*p* < 0.05, ∗∗*p* < 0.01, ∗∗∗*p* < 0.001. G1-G6 are denoted as Control, NIR Laser, MO@MB, MO@MB + NIR Laser, MOMn@MB and MOMn@MB + NIR Laser groups, respectively.

We have previously evaluated the immunotherapeutic efficacy of nanoparticles against both primary and metastatic tumors *in vivo*. After the initial treatment, immune memory confers long-term protective effects, which are significance for preventing postoperative tumor recurrence in gastric cancer. Therefore, we re-implanted the same tumor into the right hind limb subsequent to remove the primary tumor from the left hind limb with different treatments. Subsequently, we monitored tumor growth to simulate recurrence and evaluate long-term immune memory effects (Fig. 7a).

In the tumor re-challenge experiment, compared with the rapid tumor growth in the G1 group, all treatment groups except the G2 group displayed an inhibitory effect against re-inoculated tumors. Among them, G6 group demonstrated the most pronounced suppression (Fig. 7b–d, Fig. S31). This finding suggests that MOMn@MB + NIR Laser effectively elicited a potent anti-tumor immune response and significantly inhibited tumor recurrence. To further investigate the immunological mechanisms of this protective effect and assess the immune memory response mediated by MOMn@MB combined with NIR Laser, we analyzed the proportions of central memory T cells (Tcm, CD3^+^CD8^+^CD44^+^CD62L+) and effector memory T cells (Tem, CD3^+^CD8^+^CD44^+^CD62L-) in the spleens of mice subjected to different treatments prior to secondary tumor inoculation. The results indicated that, in comparison with other groups, the G6 group exhibited a significantly elevated frequency of both Tcm and Tem (Fig. 7e–g). These findings suggest that MOMn@MB + NIR Laser can induce sustained immune responses, consequently inhibiting tumor recurrence.

## 4 Conclusions

In summary, we constructed a nanoparticle MOMn@MB, which consists of MPDA, OXP, MnO_2_, and MFC cell membranes. Among these components, MPDA exhibits excellent mild photothermal properties, MnO_2_ can convert the abundant H_2_O_2_ into O_2_ in the TME due to its CAT activity, thereby alleviating tumor hypoxia and inhibiting the HIF-1α/glycolysis axis to reduce ATP production. This ultimately enhances mPTT by lowering HSP expression. Additionally, the surface modification of nanoparticles with cell membranes improves the targeting ability through homologous binding with tumor cells. ROS generated by mPTT, OXP, and MnO_2_ mediated Fenton-like reactions can induce ICD and release DAMPs. Furthermore, mPTT, loading of OXP, and ROS production cause DNA damage, activating the cGAS-STING pathway and triggering an antitumor immune response. MOMn@MB serves as a promising nanoparticle that not only addresses the heat resistance in mPTT by interfering tumor glycolysis, but also enhances the targeting capability of the nanoparticles. By inducing ICD and activating the cGAS-STING pathway, this nanoparticle triggers a robust tumor immune response, significantly inhibiting tumor growth, preventing recurrence and distant metastasis. The development of MOMn@MB has made it possible to combine chemotherapy, enhanced mPTT, and tumor immunotherapy, offering broad prospects for the treatment of gastric cancer.

## CRediT authorship contribution statement

**Henan Xu:** Writing – review & editing, Writing – original draft, Visualization, Validation, Software, Methodology, Investigation, Formal analysis, Conceptualization. **Yuxin Jiang:** Visualization, Validation, Investigation, Data curation. **Ruohao Zhang:** Visualization, Software, Formal analysis, Data curation. **Daguang Wang:** Writing – review & editing, Supervision, Resources, Project administration, Funding acquisition. **Jing Feng:** Writing – review & editing, Supervision, Resources, Funding acquisition, Conceptualization. **Hongjie Zhang:** Writing – review & editing, Supervision, Project administration, Conceptualization.

## Declaration of generative AI and AI-assisted technologies in the writing process

During the preparation of this work the author didn't use AI or AI-assisted technology.

## Declaration of competing interest

The authors declare that they have no known competing financial interests or personal relationships that could have appeared to influence the work reported in this paper.

## Data Availability

All data needed to support the conclusions in the paper are presented in the manuscript and the Electronic Supplementary Material.

## References

[1] Bray F., Laversanne M., Sung H., Ferlay J., Siegel R.L., Soerjomataram I., Jemal A. (2024). Global cancer statistics 2022: GLOBOCAN estimates of incidence and mortality worldwide for 36 cancers in 185 countries. CA Cancer J. Clin..

[2] Inoue M. (2024). Epidemiology of gastric cancer-changing trends and global disparities. Cancers.

[3] Sundar R., Nakayama I., Markar S.R., Shitara K., van Laarhoven H.W.M., Janjigian Y.Y., Smyth E.C. (2025). Gastric cancer. Lancet.

[4] Dilruba S., Kalayda G.V. (2016). Platinum-based drugs: past, present and future. Cancer Chemother. Pharmacol..

[5] Seo W.J., Kim D.-W., Lee C.M., Park J.Y., Jang Y.-J., Park J.-M., Kim J.W., Jee Y.S., Choi S.I., Oh S.C., Kim J.-H. (2025). Intraperitoneal paclitaxel with systemic S-1 plus oxaliplatin for advanced or recurrent gastric cancer with peritoneal metastasis: a single-arm, multicenter phase II clinical trial. Eur. J. Surg. Oncol..

[6] Li Y., Zhu X., Dong Y., Yang Y., Shen D., Li Z., Li R., Dang X., Qin Z., Fan K. (2024). Nanomedicine‐enabled mild photothermal therapy strategies for enhanced antitumor treatment. Adv. NanoBiomed Res..

[7] Jiang Z., Li T., Cheng H., Zhang F., Yang X., Wang S., Zhou J., Ding Y. (2021). Nanomedicine potentiates mild photothermal therapy for tumor ablation. Asian J. Pharm. Sci..

[8] Wang X., Xie L., Zhu L. (2021). Clinicopathological significance of HSP70 expression in gastric cancer: a systematic review and meta-analysis. BMC Gastroenterol..

[9] Shen G., Liu S., Cao Y., Chen Z., Wang G., Yu L., Sun L., Ran Y. (2025). HSP90 co-regulates the formation and nuclear distribution of the glycolytic output complex to promote resistance and poor prognosis in gastric cancer patients. J. Transl. Med..

[10] Zhao K., Zhou G., Liu Y., Zhang J., Chen Y., Liu L., Zhang G. (2023). HSP70 family in cancer: signaling mechanisms and therapeutic advances. Biomolecules.

[11] Kunachowicz D., Król-Kulikowska M., Raczycka W., Sleziak J., Błażejewska M., Kulbacka J. (2024). Heat shock proteins, a double-edged sword: significance in cancer progression, chemotherapy resistance and novel therapeutic perspectives. Cancers.

[12] Qiao Q., Hu S., Wang X. (2024). The regulatory roles and clinical significance of glycolysis in tumor. Cancer Commun..

[13] Kierans S.J., Taylor C.T. (2020). Regulation of glycolysis by the hypoxia‐inducible factor (HIF): implications for cellular physiology. J. Physiol..

[14] Martínez-Reyes I., Chandel N.S. (2021). Cancer metabolism: looking forward. Nat. Rev. Cancer.

[15] Yang Q., Lei X., He J., Peng Y., Zhang Y., Ling R., Wu C., Zhang G., Zheng B., Chen X., Zou B., Fu Z., Zhao L., Liu H., Hu Y., Yu J., Li F., Ye G., Li G. (2023). N4‐Acetylcytidine drives glycolysis addiction in gastric cancer via NAT10/SEPT9/HIF‐1α positive feedback loop. Adv. Sci..

[16] Luo B., Song L., Chen L., Cai Y., Zhang M., Wang S. (2024). Ganoderic acid D attenuates gemcitabine resistance of triple-negative breast cancer cells by inhibiting glycolysis via HIF-1α destabilization. Phytomedicine.

[17] Dong S., Liang S., Cheng Z., Zhang X., Luo L., Li L., Zhang W., Li S., Xu Q., Zhong M., Zhu J., Zhang G., Hu S. (2022). ROS/PI3K/Akt and Wnt/β-catenin signalings activate HIF-1α-induced metabolic reprogramming to impart 5-fluorouracil resistance in colorectal cancer. J. Exp. Clin. Cancer Res..

[18] Zhang W., Chen J., Wei Z., Song J., Zha X., Wang D., Xu M. (2025). Advancements and challenges in immunotherapy for gastric cancer: current approaches and future directions. Front. Immunol..

[19] Engelen Y., Demuynck R., Ramon J., Breckpot K., De Smedt S.C., Lajoinie G.P.R., Braeckmans K., Krysko D.V., Lentacker I. (2025). Immunogenic cell death as interplay between physical anticancer modalities and immunotherapy. J. Contr. Release.

[20] Galluzzi L., Guilbaud E., Schmidt D., Kroemer G., Marincola F.M. (2024). Targeting immunogenic cell stress and death for cancer therapy. Nat. Rev. Drug Discov..

[21] Yu H., Liu S., Yuan Z., Huang H., Yan P., Zhu W. (2024). Targeted co-delivery of rapamycin and oxaliplatin by liposomes suppresses tumor growth and metastasis of colorectal cancer. Biomed. Pharmacother..

[22] Ran J., Liu T., Song C., Wei Z., Tang C., Cao Z., Zou H., Zhang X., Cai Y., Han W. (2023). Rhythm mild-temperature photothermal therapy enhancing immunogenic cell death response in oral squamous cell carcinoma. Adv. Healthcare Mater..

[23] Malla R., Kumari S., Ganji S.P., Srilatha M., Nellipudi H.R., Nagaraju G.P. (2024). Reactive oxygen species of tumor microenvironment: harnessing for immunogenic cell death. Biochim. Biophys. Acta, Rev. Cancer.

[24] Zhang C., Wang X., Du J., Gu Z., Zhao Y. (2020). Reactive oxygen species‐regulating strategies based on nanomaterials for disease treatment. Adv. Sci..

[25] Chen M., Dong C., Shi S. (2022). An overview of recent advancements on manganese-based nanostructures and their application for ROS-mediated tumor therapy. ACS Mater. Lett..

[26] Hopfner K.-P., Hornung V. (2020). Molecular mechanisms and cellular functions of cGAS-STING signalling. Nat. Rev. Mol. Cell Biol..

[27] Liang J.-L., Jin X.-K., Deng X.-C., Huang Q.-X., Zhang S.-M., Chen W.-H., Zhang X.-Z. (2024). Targeting activation of cGAS-STING signaling pathway by engineered biomaterials for enhancing cancer immunotherapy. Mater. Today.

[28] Zhang K., Qi C., Cai K. (2023). Manganese-based tumor immunotherapy. Adv. Mater..

[29] Li Q.-R., Zhang X., Zhang C., Zhang Y., Niu M.-T., Chen Z., Zhang S.-M., He J., Chen W.-H., Zhang X.-Z. (2025). Biomineralized engineered bacterial outer membrane vesicles as cGAS-STING nanoagonists synergize with lactate metabolism modulation to potentiate immunotherapy. J. Am. Chem. Soc..

[30] Liang J.-L., Jin X.-K., Zhang S.-M., Huang Q.-X., Ji P., Deng X.-C., Cheng S.-X., Chen W.-H., Zhang X.-Z. (2023). Specific activation of cGAS-STING pathway by nanotherapeutics-mediated ferroptosis evoked endogenous signaling for boosting systemic tumor immunotherapy. Sci. Bull..

[31] Gong F., Yang N., Wang X., Zhao Q., Chen Q., Liu Z., Cheng L. (2020). Tumor microenvironment-responsive intelligent nanoplatforms for cancer theranostics. Nano Today.

[32] Pang M., Long G., Jiang S., Ji Y., Han W., Wang B., Liu X., Xi Y. (2015). One pot low-temperature growth of hierarchical δ-MnO2 nanosheets on nickel foam for supercapacitor applications. Electrochim. Acta.

[33] Li Y., Xu Z., Wang D., Zhao J., Zhang H. (2017). Snowflake-like core-shell α-MnO2@δ-MnO2 for high performance asymmetric supercapacitor. Electrochim. Acta.

[34] Zhang R., Yu J., Ma K., Ma Y., Wang Z. (2020). Synergistic chemo-photothermal antibacterial effects of polyelectrolyte-functionalized gold nanomaterials. ACS Appl. Bio Mater..

[35] Moura J.P., Oliveira P.J., Urbano A.M. (2025). Mitochondrial classic metabolism and its often-underappreciated facets. Biochim. Biophys. Acta, Mol. Basis Dis..

[36] Liu K., Jing M.J., Liu C., Yan D.Y., Ma Z., Wang C., Deng Y., Liu W., Xu B. (2019). Effect of trehalose on manganese-induced mitochondrial dysfunction and neuronal cell damage in mice. Basic Clin. Pharmacol. Toxicol..

[37] Xie Q., Sun T., Zhang L., Gong M., Zhang W., Liu X., Zhao Y., Wang M., Yang X., Zhang Z., Liu G., Zhou C., Zhang D. (2025). Responsive plasmonic hybrid nanorods enables metabolism reprogramming via cuproptosis-photothermal combined cancer therapy. Biomaterials.

