# Supplementary material for: Multiple-pathway cGAS-STING activation with enhanced mild photothermal therapy through glycolysis regulation for boosting gastric cancer immunotherapy
Source: Mater Today Bio. 2026 Jan 9;37:102790. doi: 10.1016/j.mtbio.2026.102790 (PMC12857272; doi:10.1016/j.mtbio.2026.102790)
Supplement: Multimedia component 1 [file mmc1.doc]

**Supporting Information**

**Multiple-pathway cGAS-STING activation with enhanced mild photothermal therapy through glycolysis regulation for boosting gastric cancer immunotherapy**

Henan Xu^a^, Yuxin Jiang^b^, Ruohao Zhang^c^, Daguang Wang^a*^, Jing Feng^c*^, Hongjie Zhang^c*^

^a^ *Department of Gastrocolorectal Surgery, General Surgery Center, The First Hospital of Jilin University, Changchun 130021, PR China*

^b^ *College of Pharmacy, Changchun University of Chinese Medicine, Changchun 130117, PR China*

^c^ *State Key Laboratory of Rare Earth Resource Utilization, Changchun Institute of Applied Chemistry, Chinese Academy of Sciences, Changchun 130022, PR China*

*Corresponding authors (email: Daguang Wang: dgwang@jlu.edu.cn; Jing Feng: fengj@ciac.ac.cn; Hongjie Zhang: hongjie@ciac.ac.cn)

**Supplementary Figures**


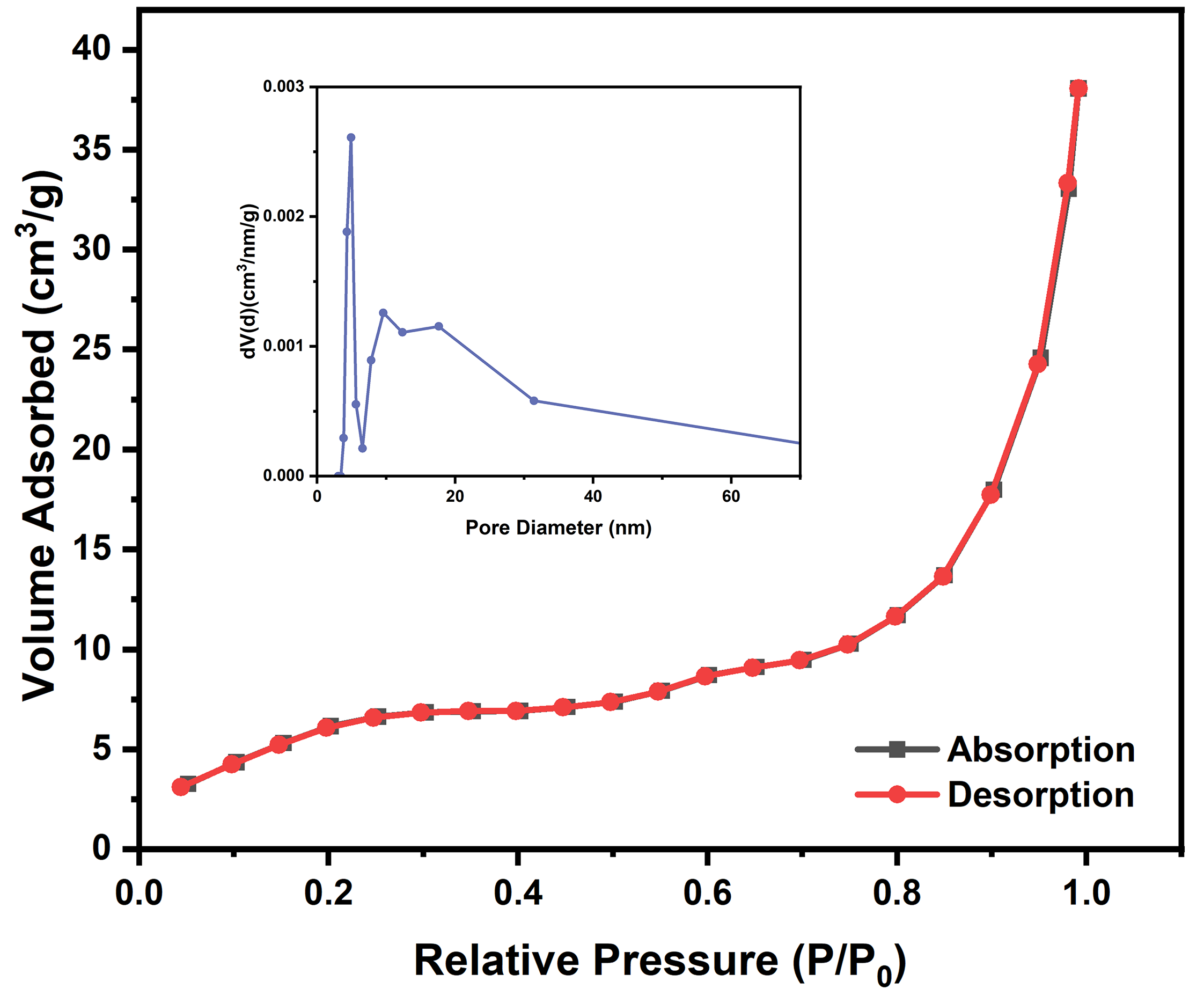


**Figure S1** Nitrogen adsorption-desorption isotherms of MPDA nanoparticles. Inset: Pore size distribution of MPDA nanoparticles.


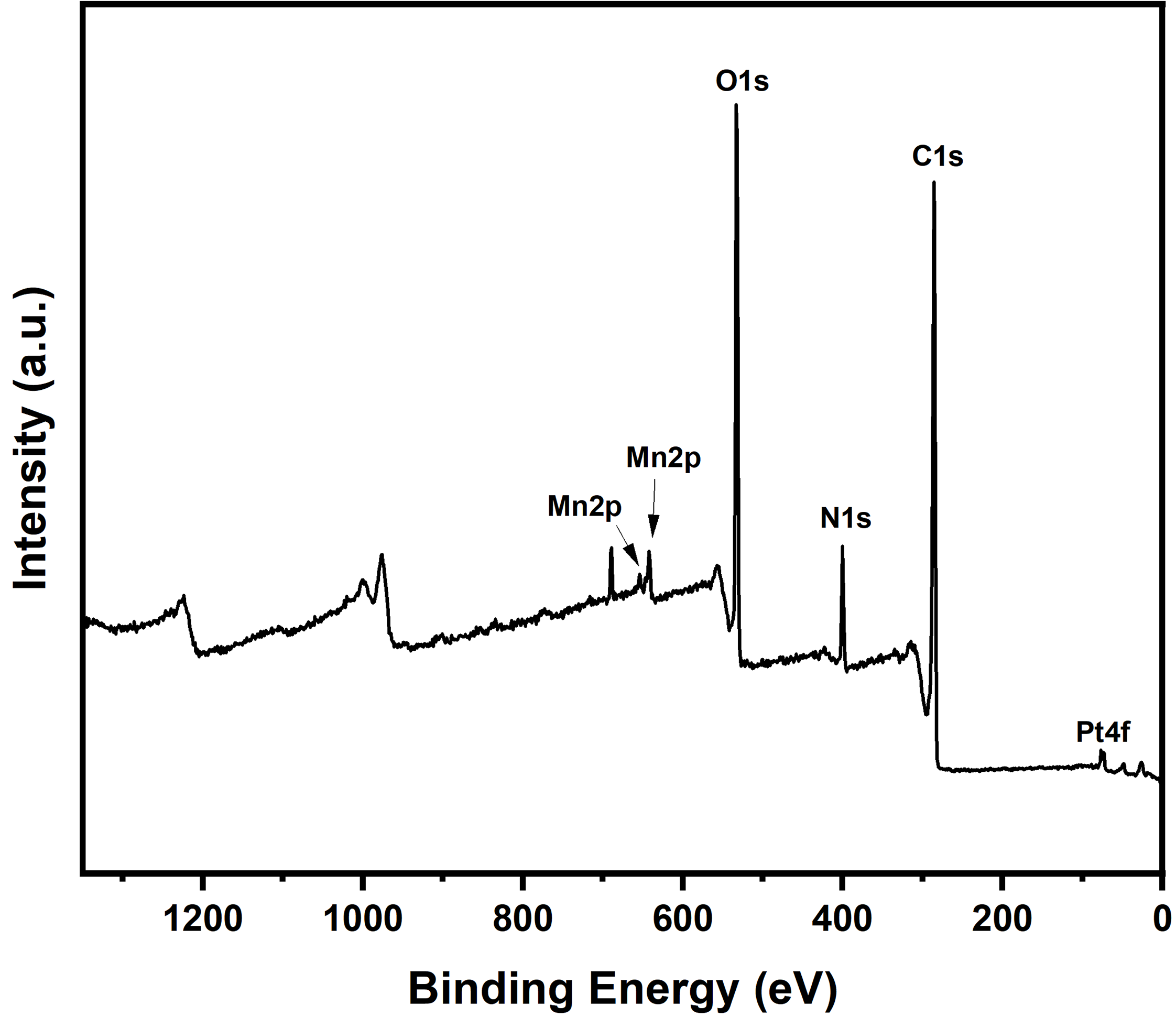


**Figure S2** XPS spectrum of MOMn.


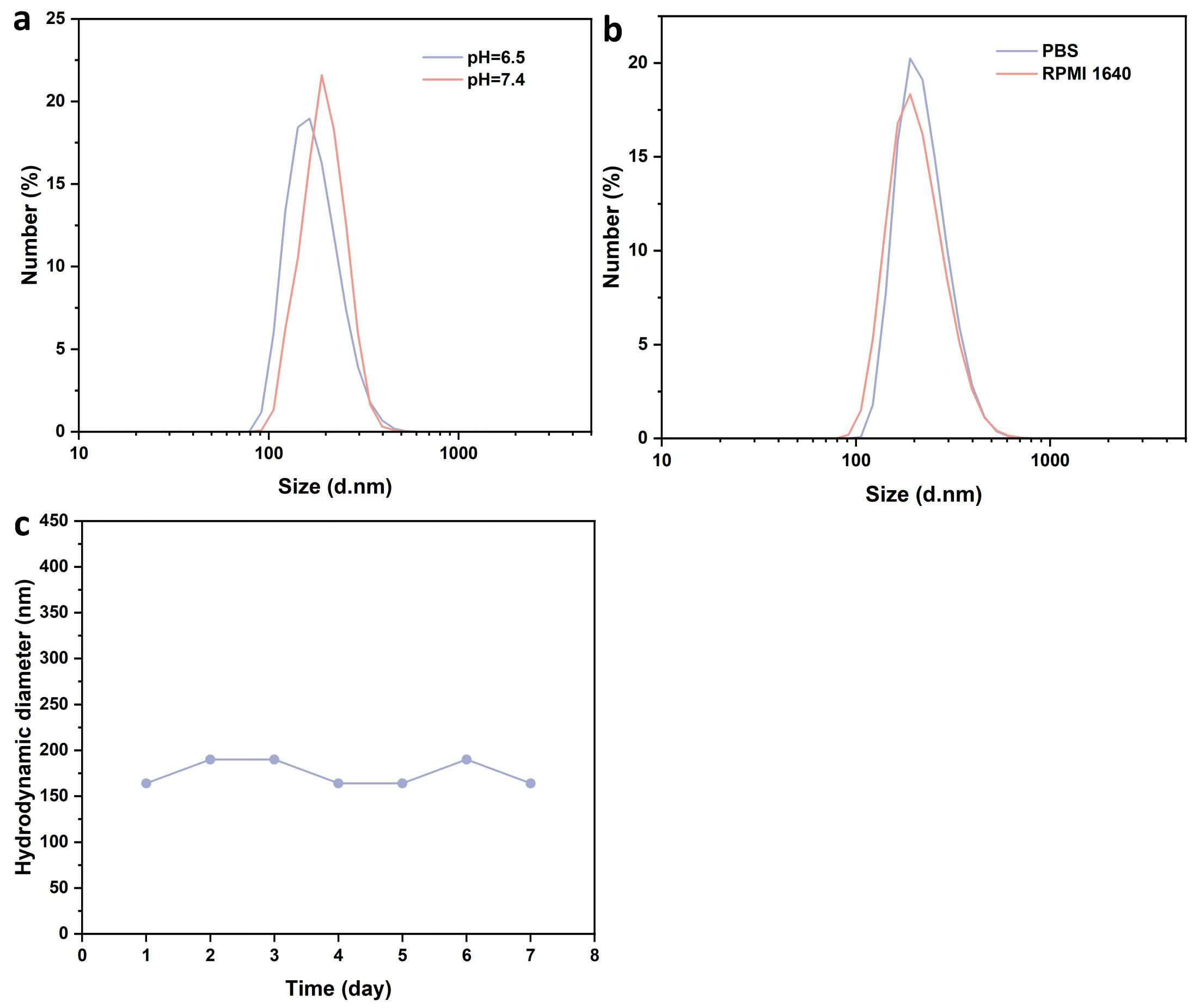


**Figure S3** Hydrodynamic size of MOMn@MB at (a) different pH (6.5 and 7.4), and in (b) different solvents (PBS and RPMI 1640 medium containing 10% FBS). (c) Hydrodynamic size changes of MOMn@MB in the H_2_O for 7 days.

**Table S1** Photothermal conversion efficiency of MPDA@MB and MOMn@MB.

| Sample | *τ*_s_ | *hA* | *Q* | *Q*_dis_ | *η* | R^2^ ***^a-^******^f^*** |
| --- | --- | --- | --- | --- | --- | --- |
| MPDA@MB | 179.1976 | 0.0047 | 0.1875 | 0.0300 | 0.3528 | 0.9961 |
|  | 189.5359 | 0.0044 | 0.1773 | 0.0284 | 0.3335 | 0.9977 |
|  | 188.4440 | 0.0045 | 0.1783 | 0.0285 | 0.3355 | 0.9986 |
|  | 184.5456 | 0.0046 | 0.1821 | 0.0291 | 0.3426 | 0.9986 |
| MOMn@MB | 195.4677 | 0.0043 | 0.1818 | 0.0279 | 0.3419 | 0.9987 |
|  | 187.4121 | 0.0045 | 0.1896 | 0.0291 | 0.3567 | 0.9985 |
|  | 189.7876 | 0.0044 | 0.1872 | 0.0288 | 0.3522 | 0.9977 |
|  | 189.3444 | 0.0044 | 0.1877 | 0.0288 | 0.3530 | 0.9981 |

**(a)** *τ*_s_: characteristic thermal time constant

**(b)** *h*: heat transfer coefficient, *A*: surface area of sample container

**(c)** *Q*: energy input to sample and sample container

**(d)** *Q*_dis_: baseline energy input for solvent and sample container

**(e)** *η*: photothermal conversion efficiency

**(f)** R^2^: coefficient of determination.


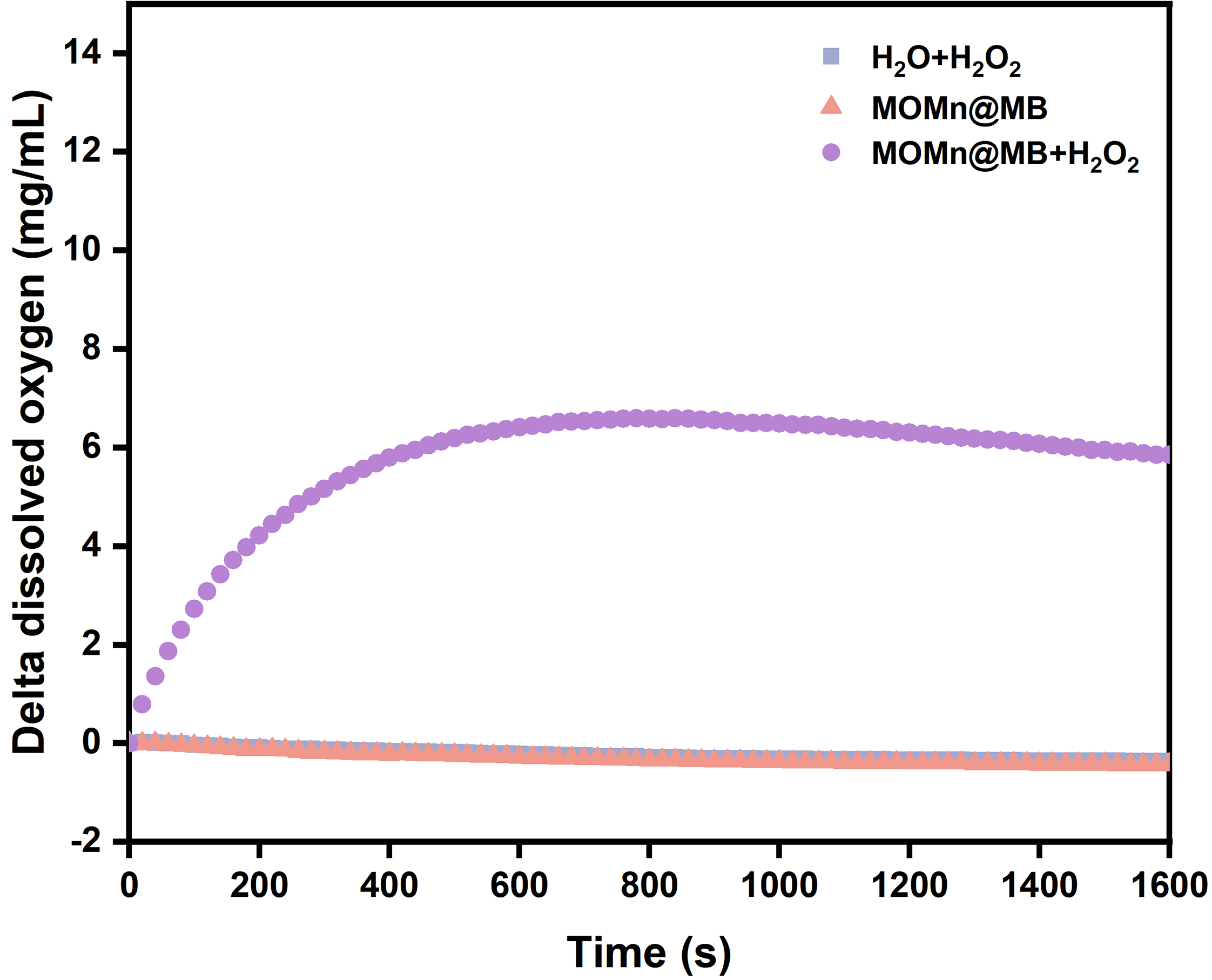


**Figure S4** Oxygen generation capability of MOMn@MB.


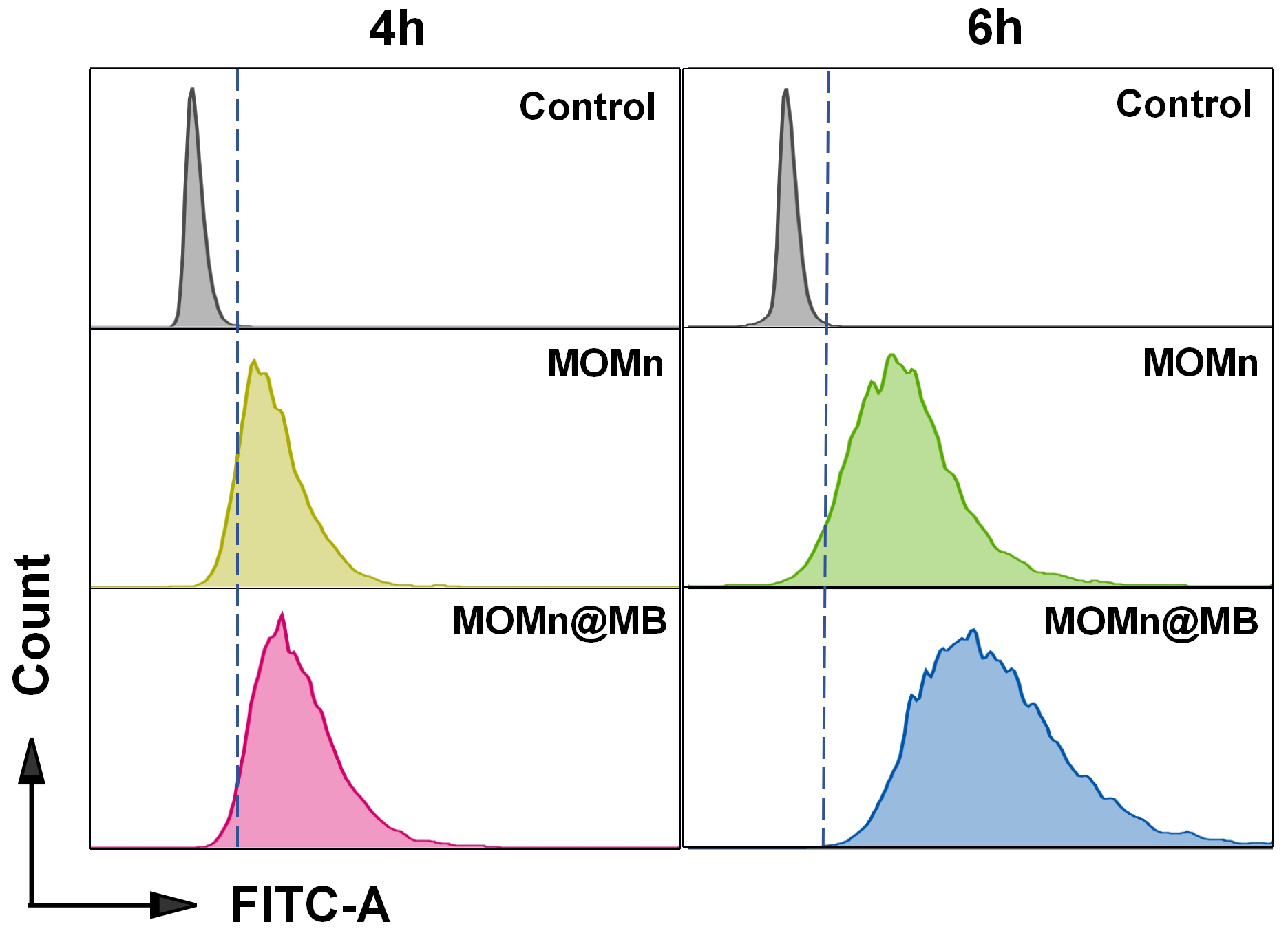


**Figure S5** Representative flow cytometry plots of MFC tumor cells co-cultured with MOMn and MOMn@MB for 4 h and 6 h.


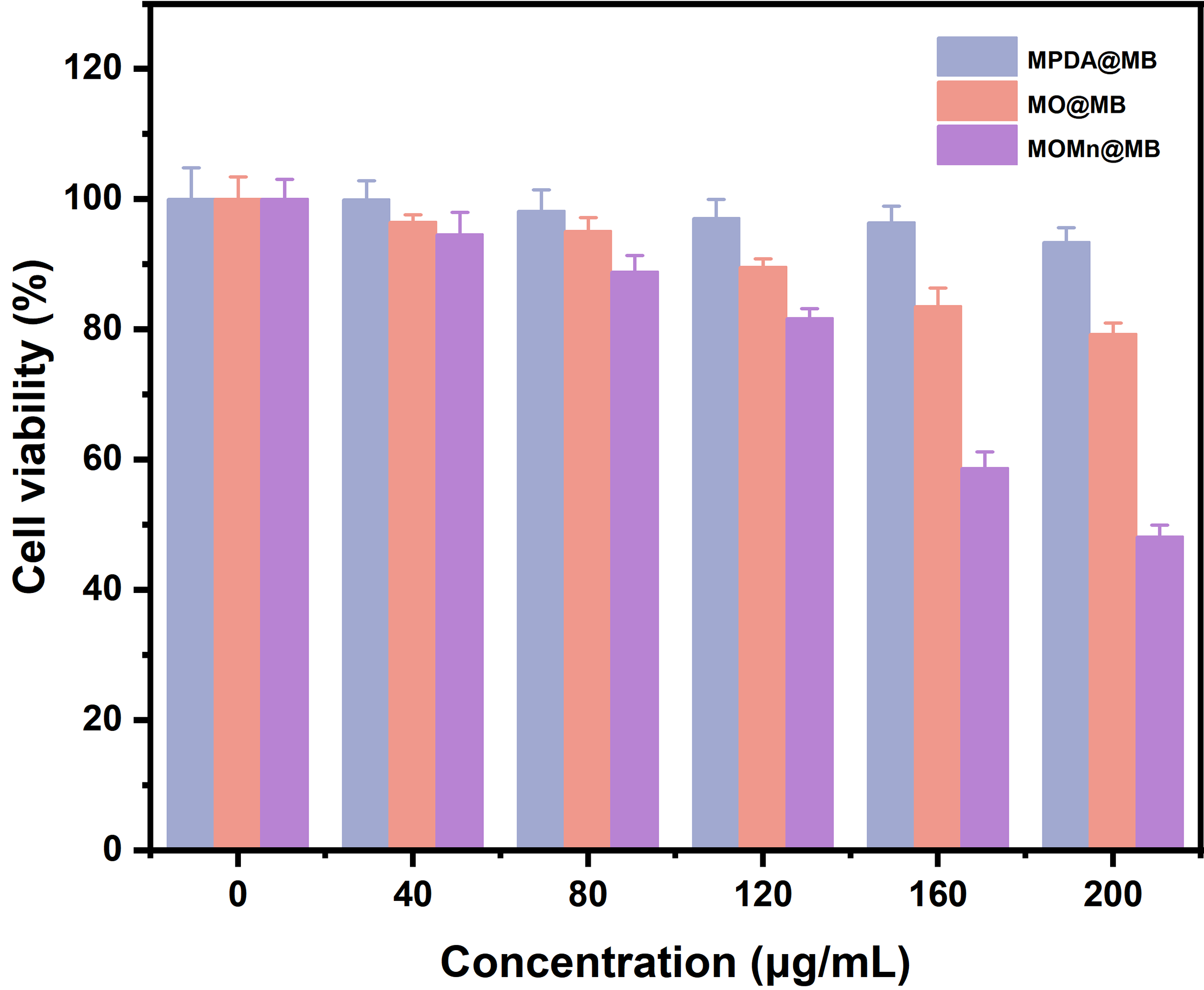


**Figure S6** Cytotoxicity of different nanoparticles on L929 cells.


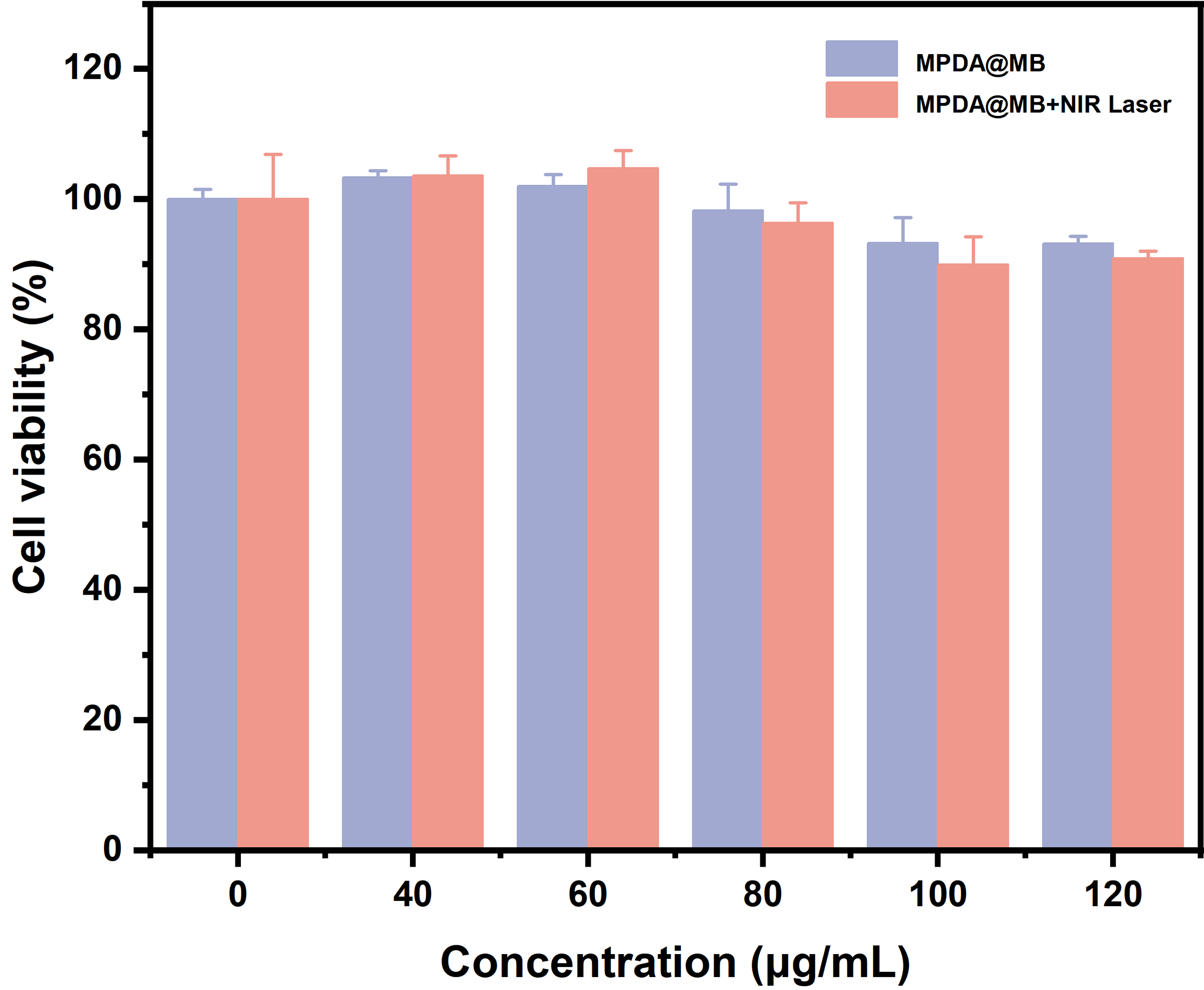


**Figure S7** Cytotoxicity assessment of MFC cells after treated with MPDA@MB and MPDA@MB + NIR Laser.


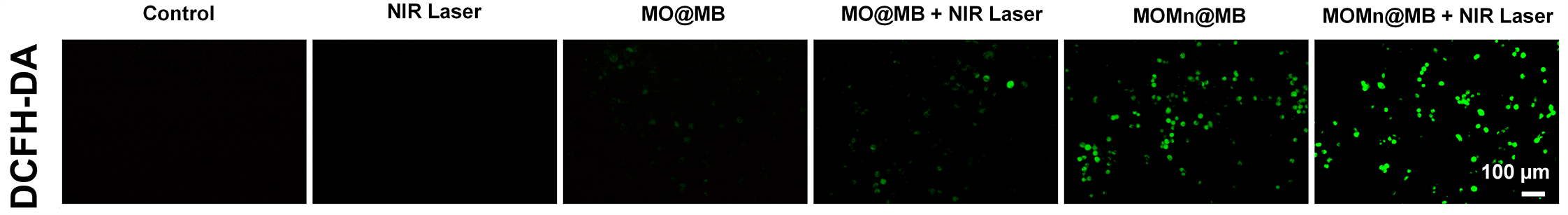


**Figure S8** ROS generation in MFC cells with different treatments.


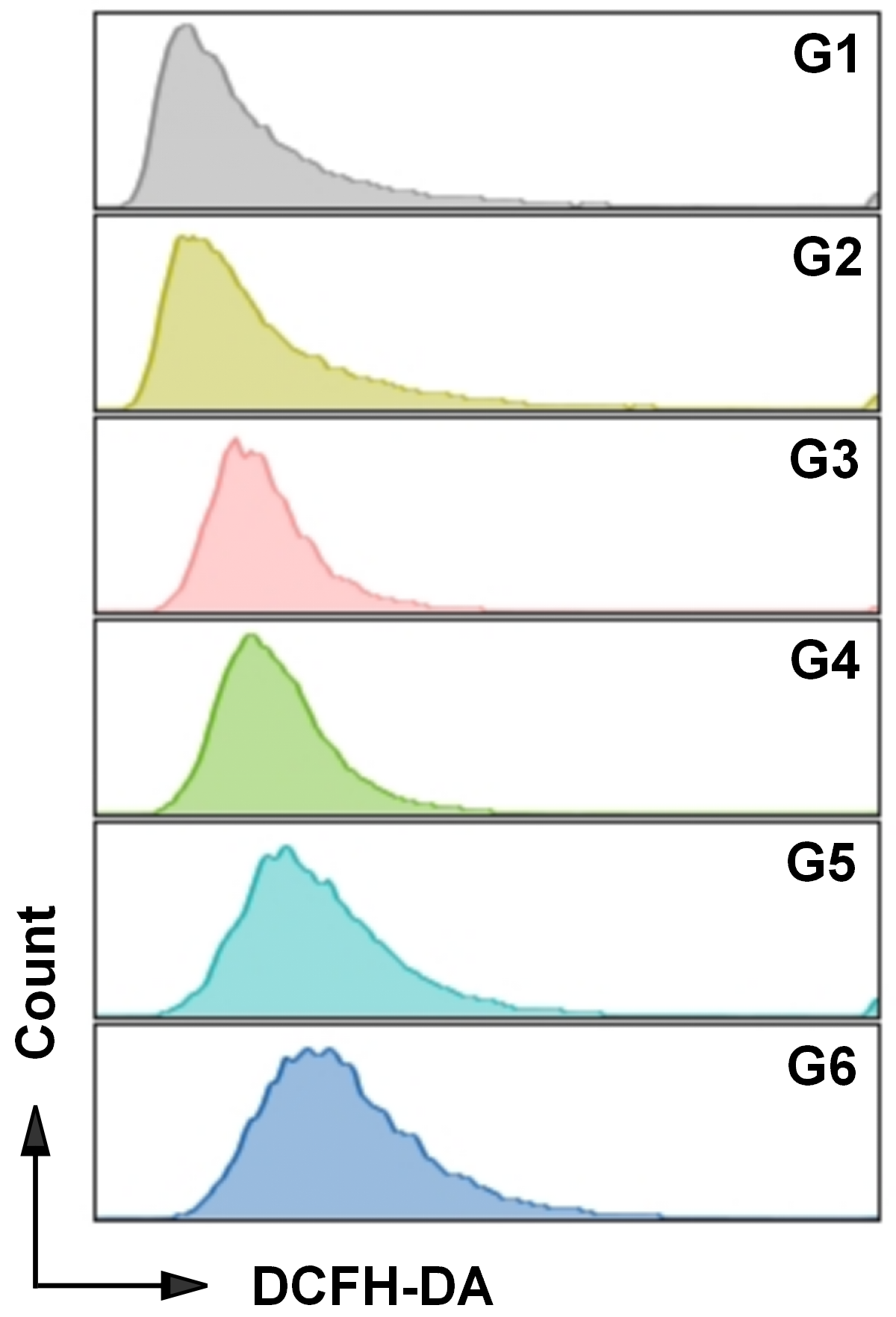


**Figure S9** Flow cytometry analysis of ROS generation in MFC tumor cells with different treatments.


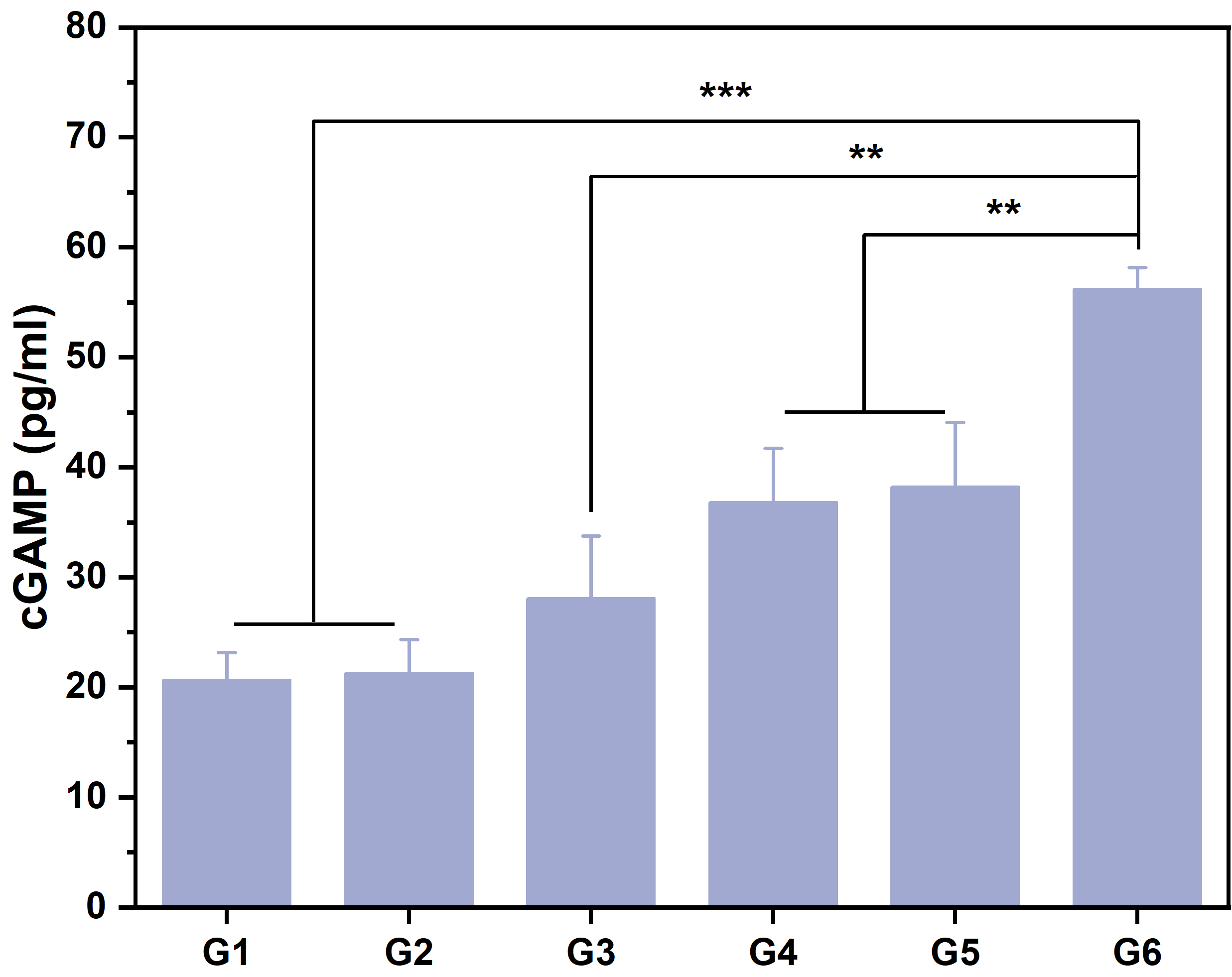


**Figure S10** Release of cGAMP in MFC cells treated with different nanoparticles. **p* < 0.05, ***p* < 0.01, ****p* < 0.001.


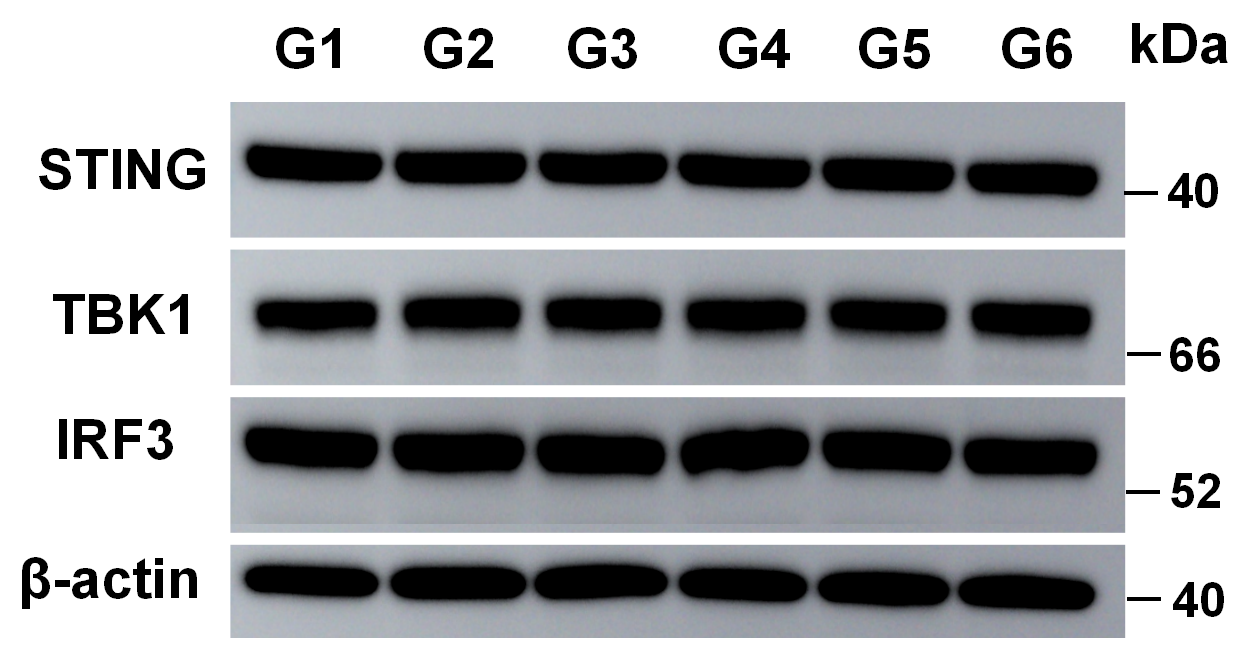


**Figure S11** Western blot analysis of cGAS-STING pathway-associated non-phosphorylated proteins in MFC tumor cells with different treatments.


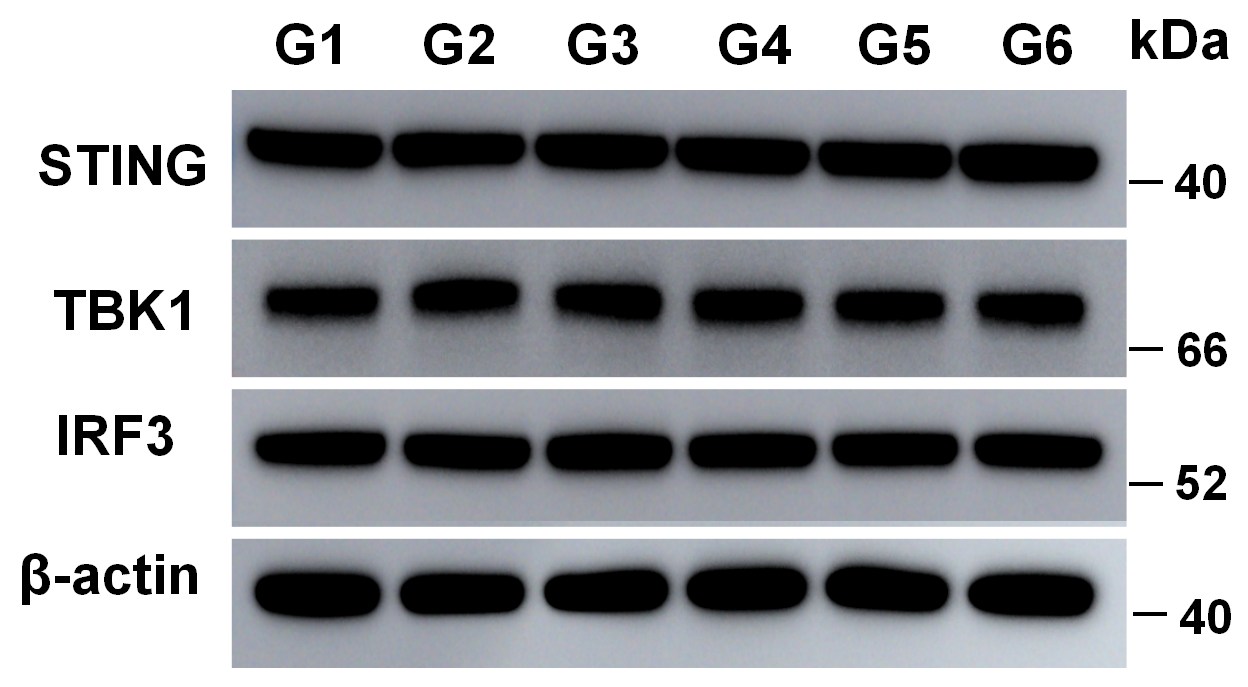


**Figure S12** Western blot analysis of cGAS-STING pathway-associated non-phosphorylated proteins in DC cells with different treatments.


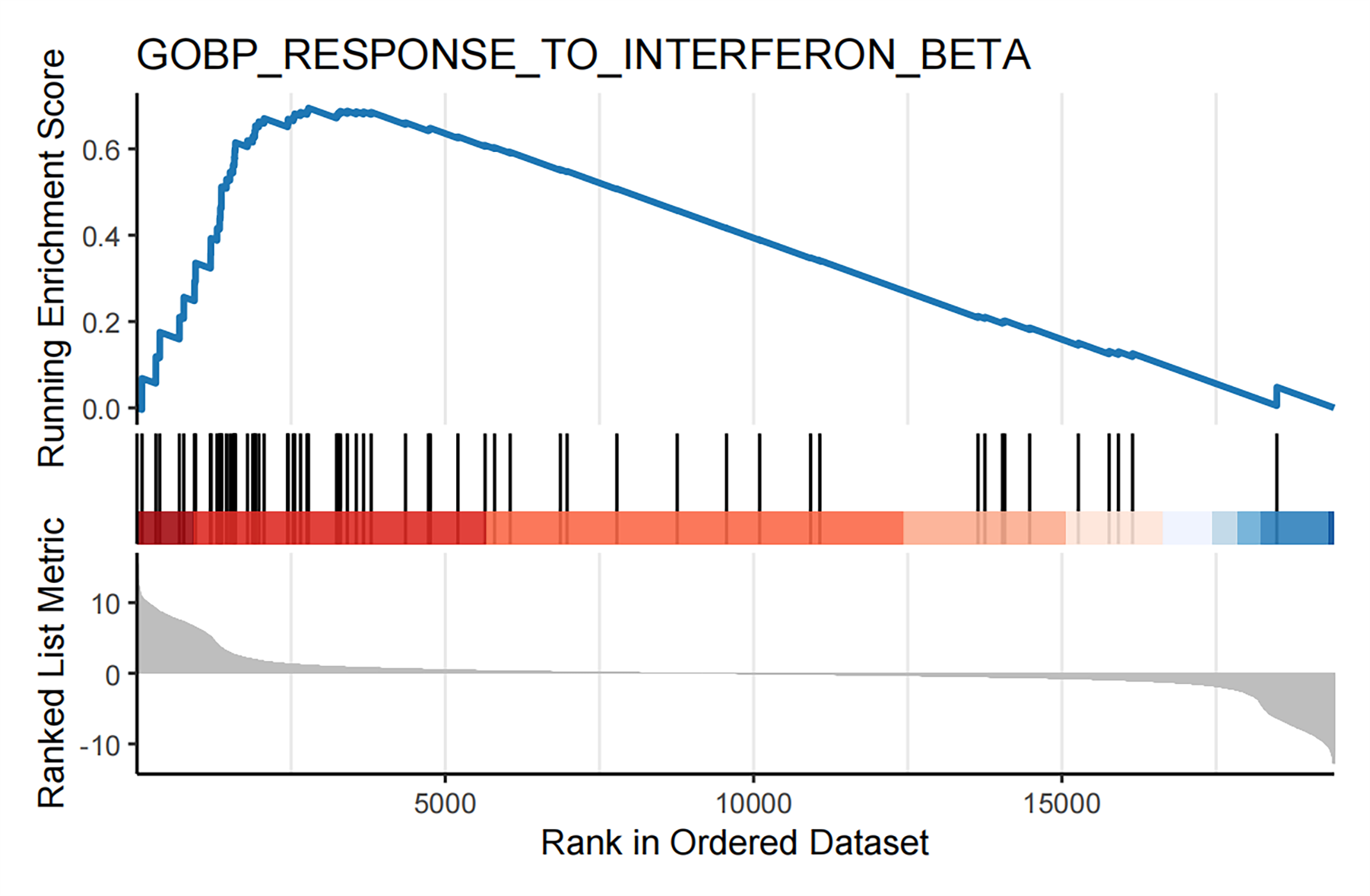


**Figure S13** GSEA enrichment analysis of "response to interferon beta".


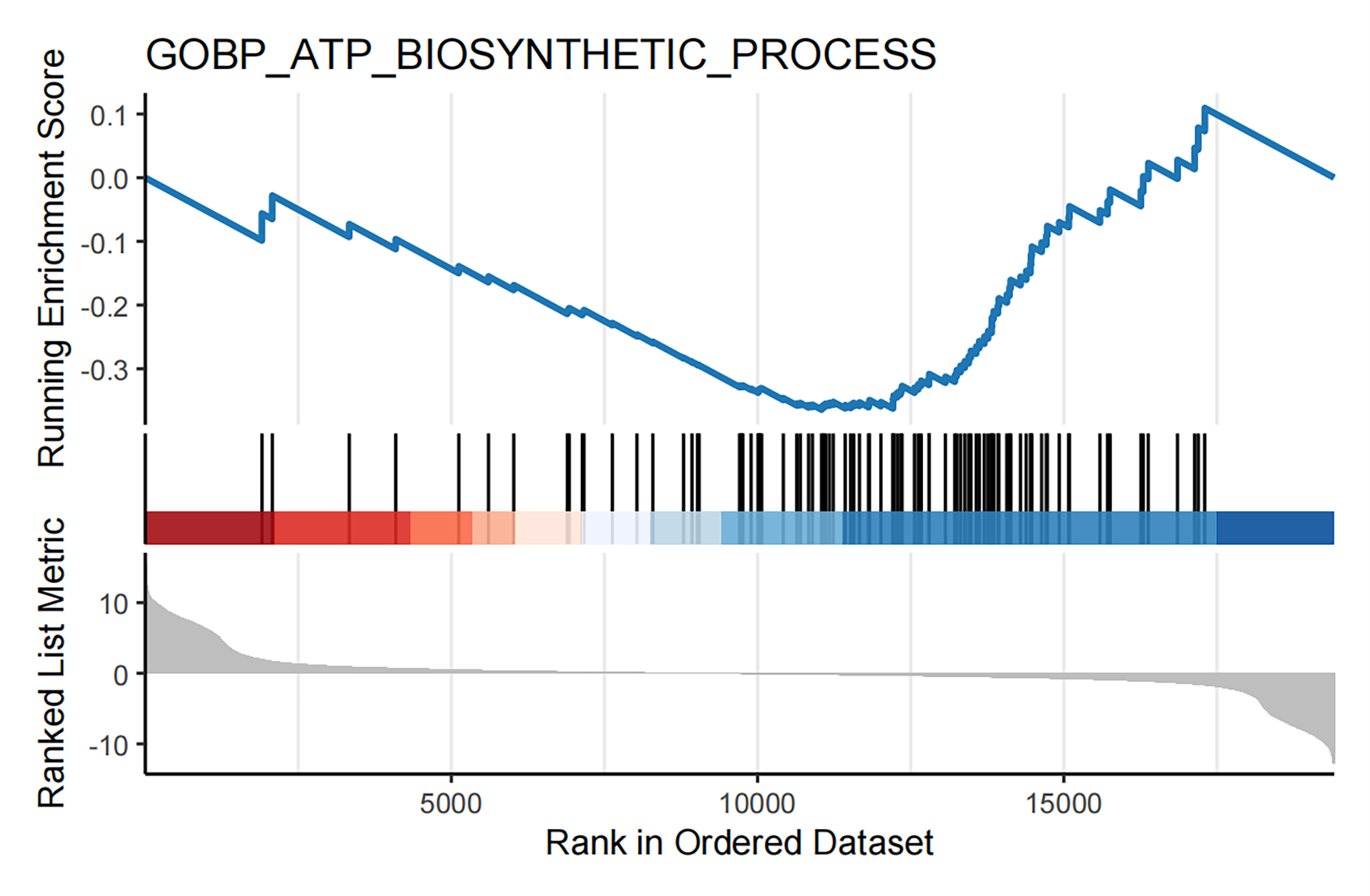


**Figure S14** GSEA enrichment analysis of "ATP biosynthetic process".


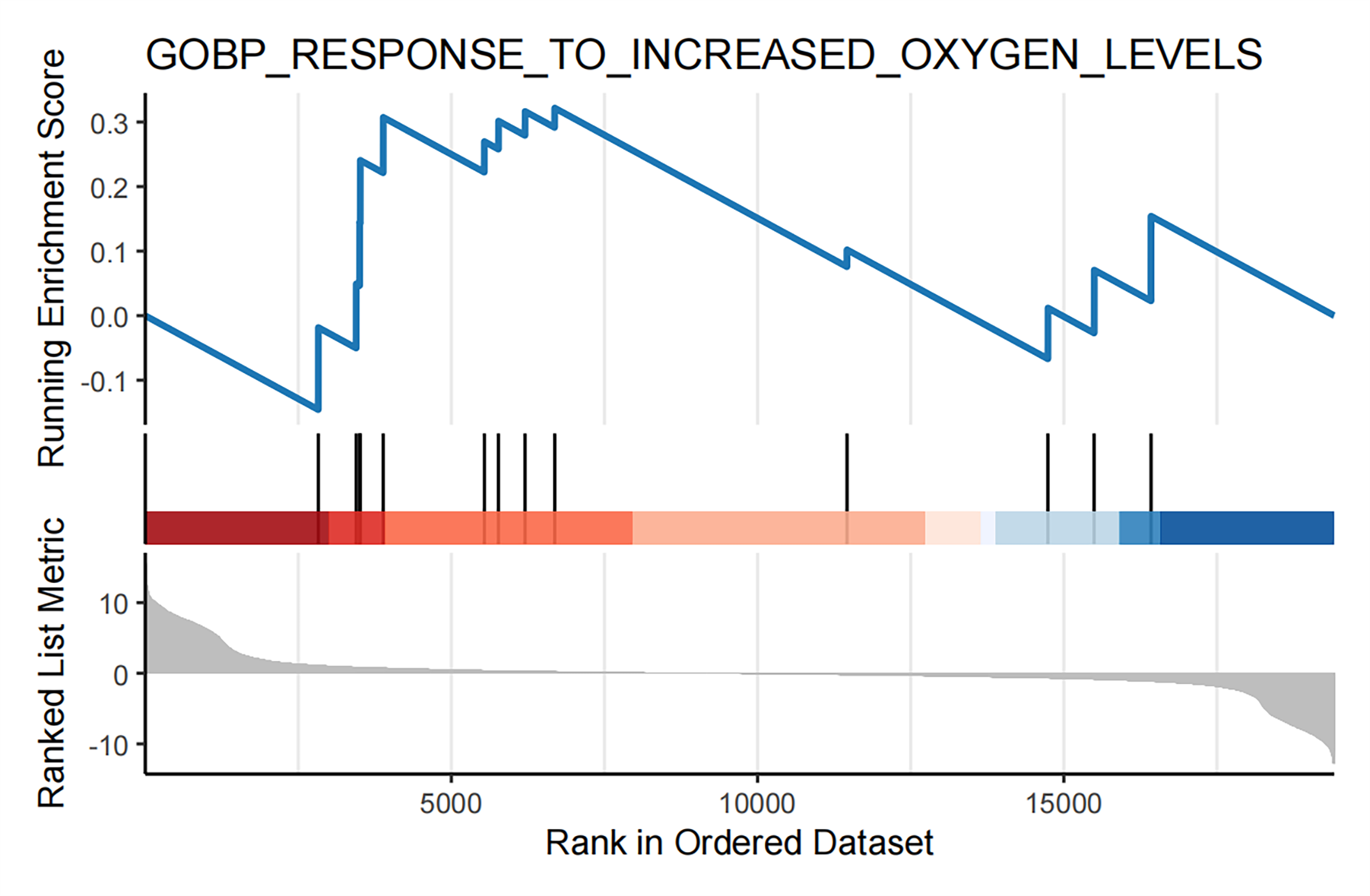


**Figure S15** GSEA enrichment analysis of "response to increased oxygen levels".


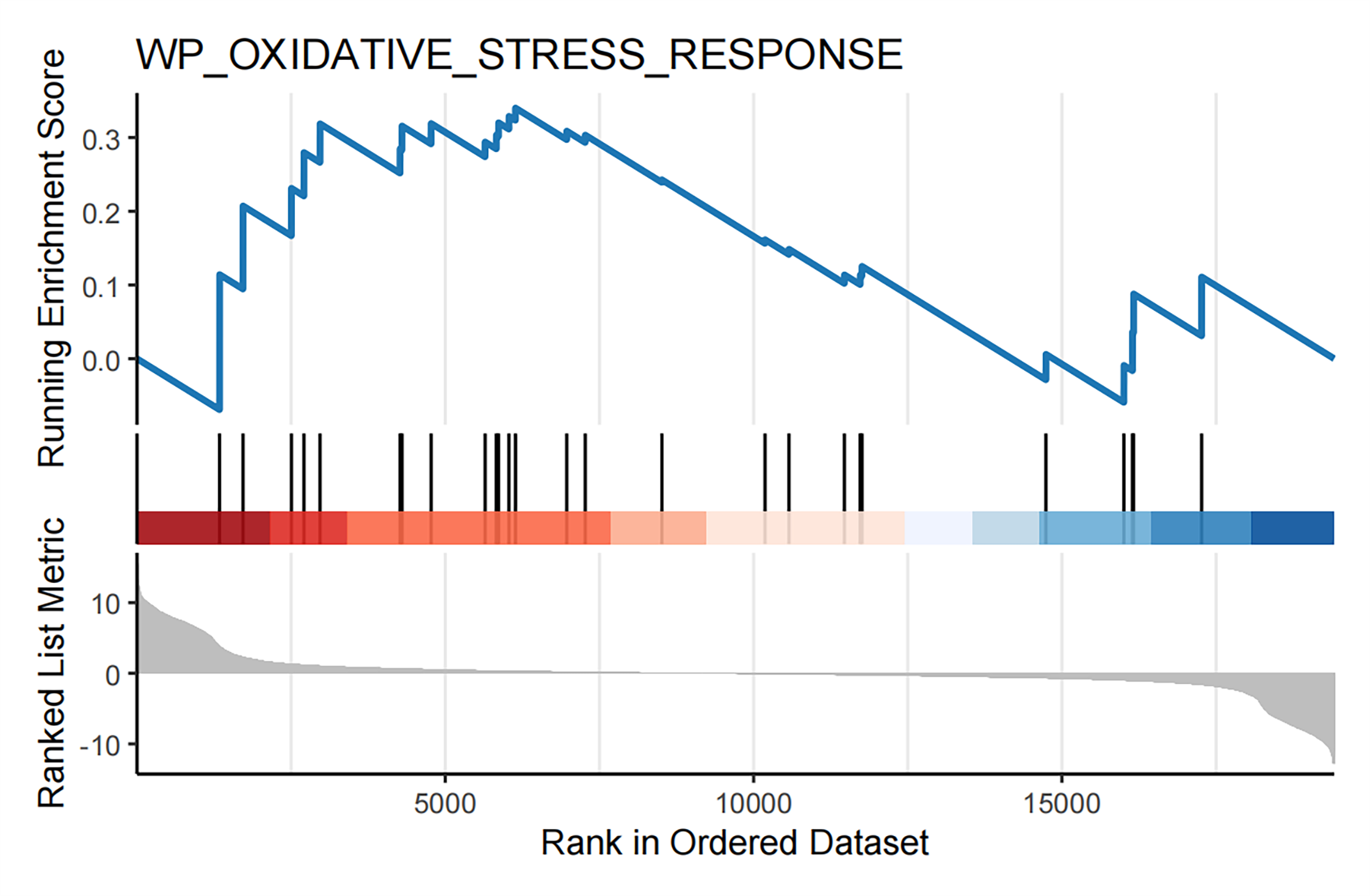


**Figure S16** GSEA enrichment analysis of "response to oxidative stress".


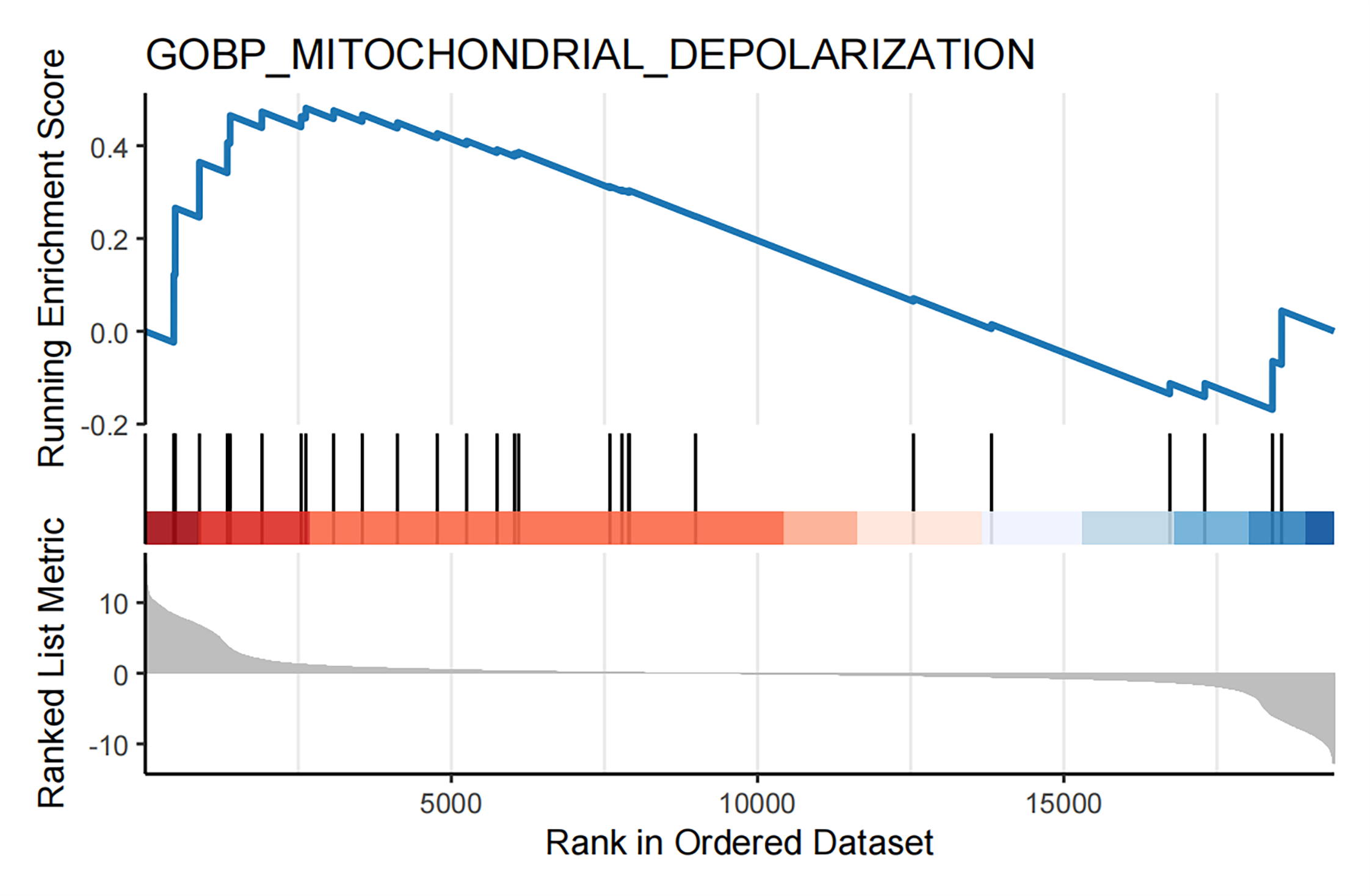


**Figure S17** GSEA enrichment analysis of "mitochondrial depolarization".


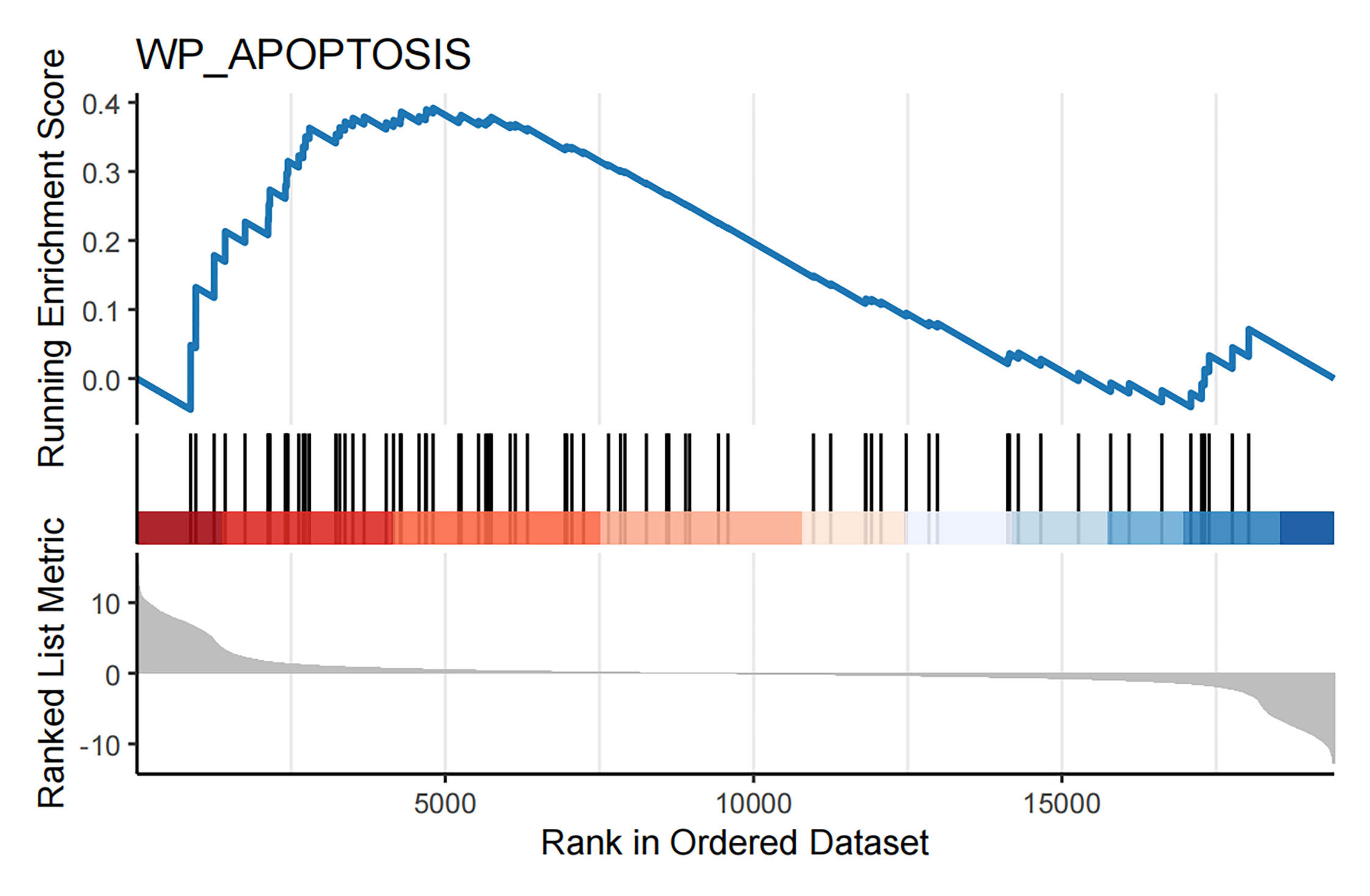


**Figure S18** GSEA enrichment analysis of "apoptosis".


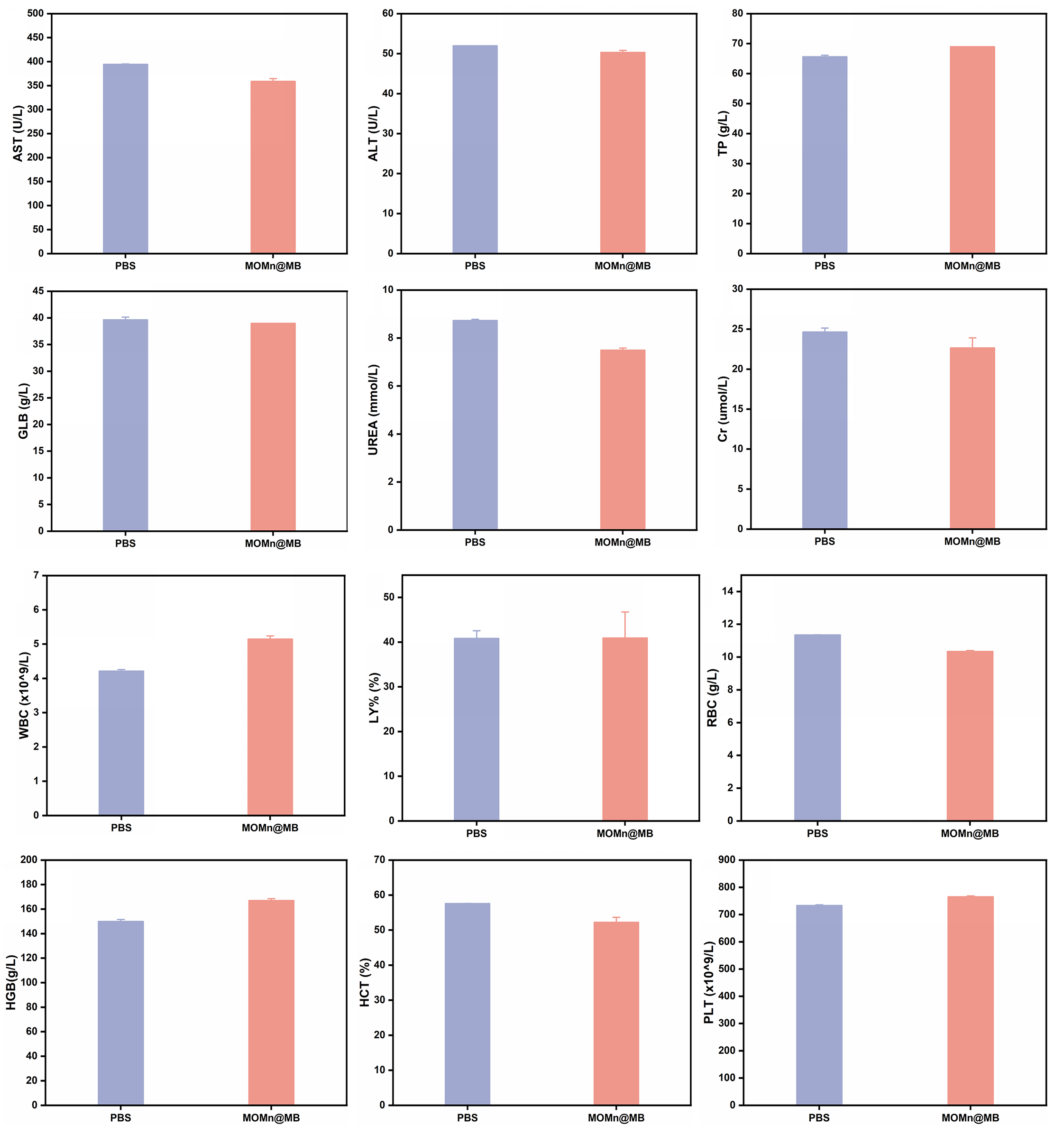


**Figure S19** Routine blood analysis after intravenous injection with PBS and MOMn@MB after 28 days. aspartate transaminase (AST), alanine transaminase (ALT), total protein (TP), globulin (GLB), creatinine (Cr), white blood cells (WBC), lymphocyte (LY), red blood cells (RBC), hemoglobin (HGB), hematocrit (HCT), platelet (PLT).


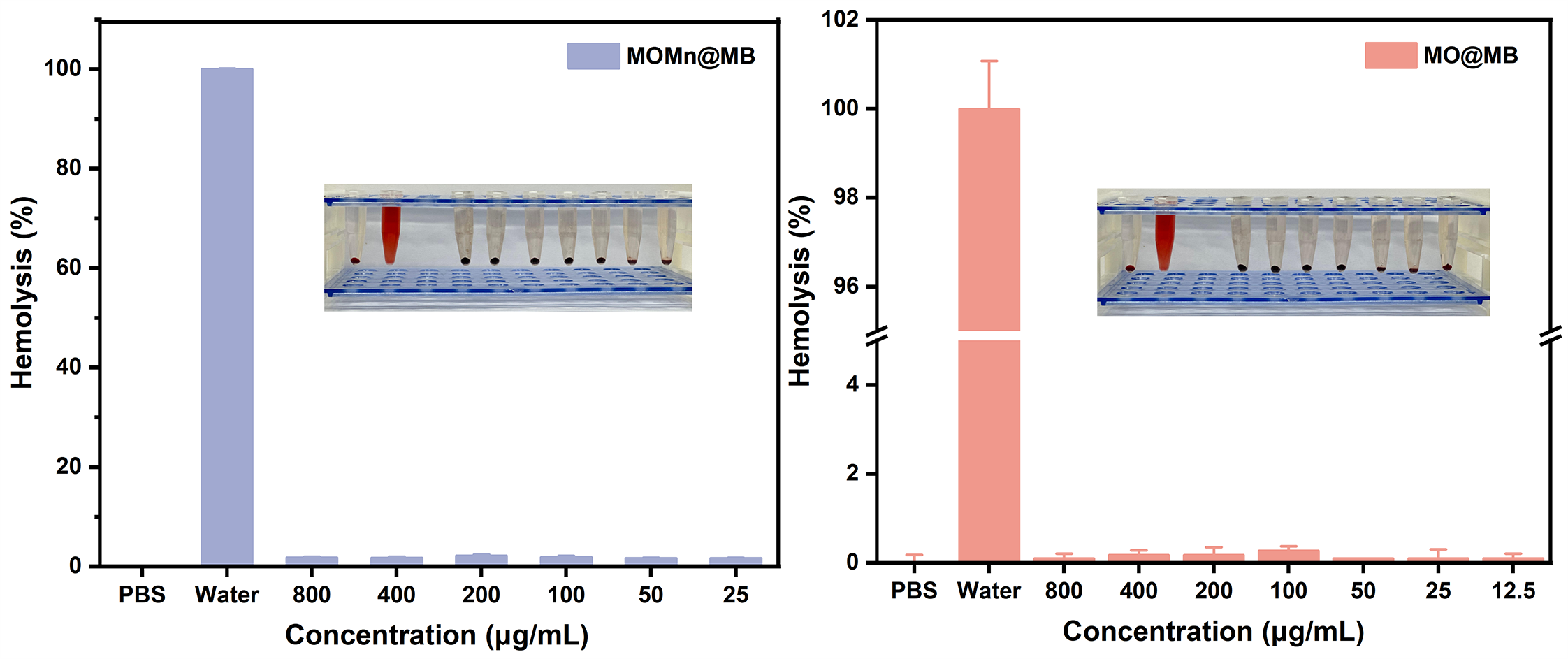


**Figure S20** Hemolysis rates of MOMn@MB and MO@MB.


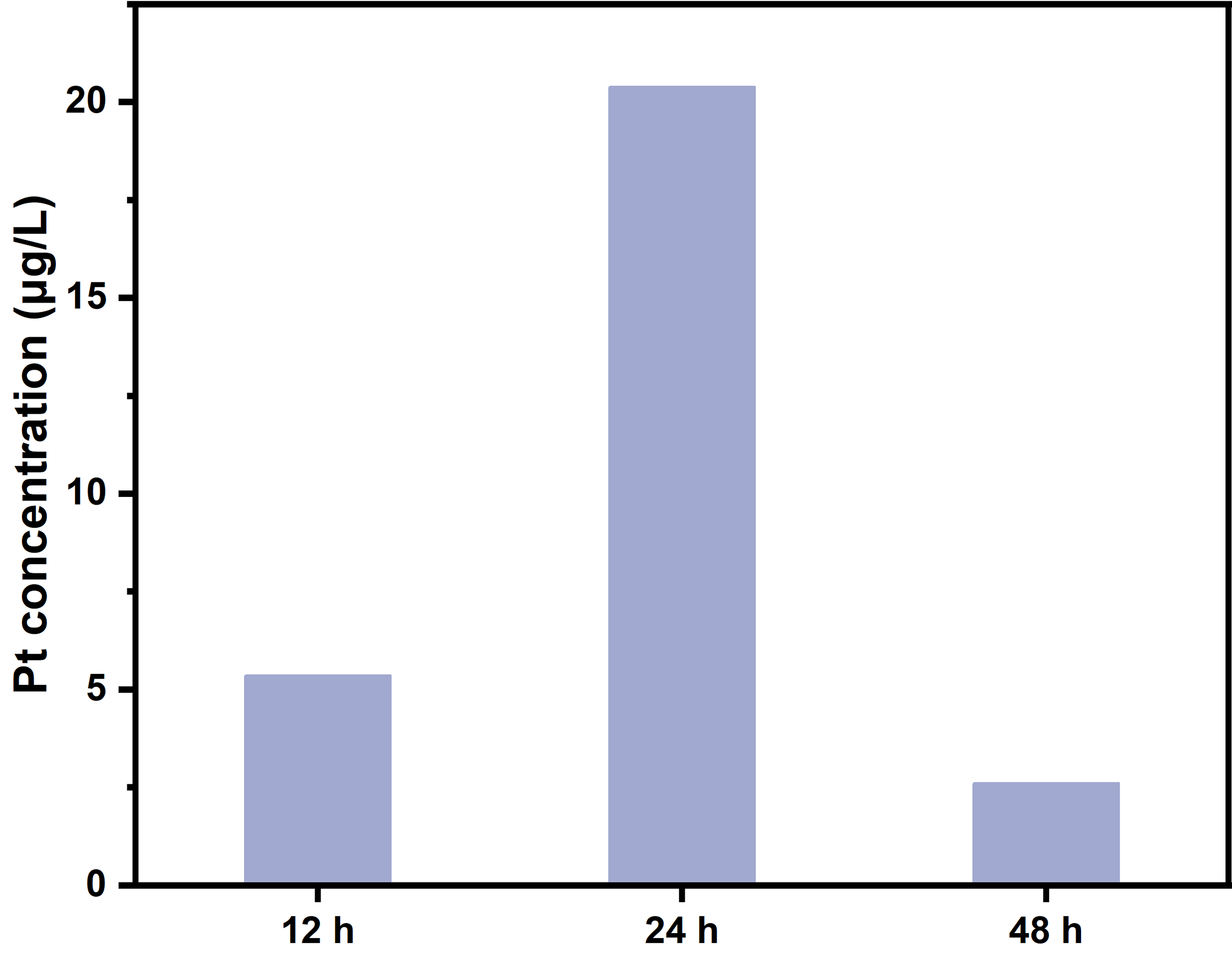


**Figure S21** The accumulation concentration of MOMn@MB in tumor tissues by ICP-MS.


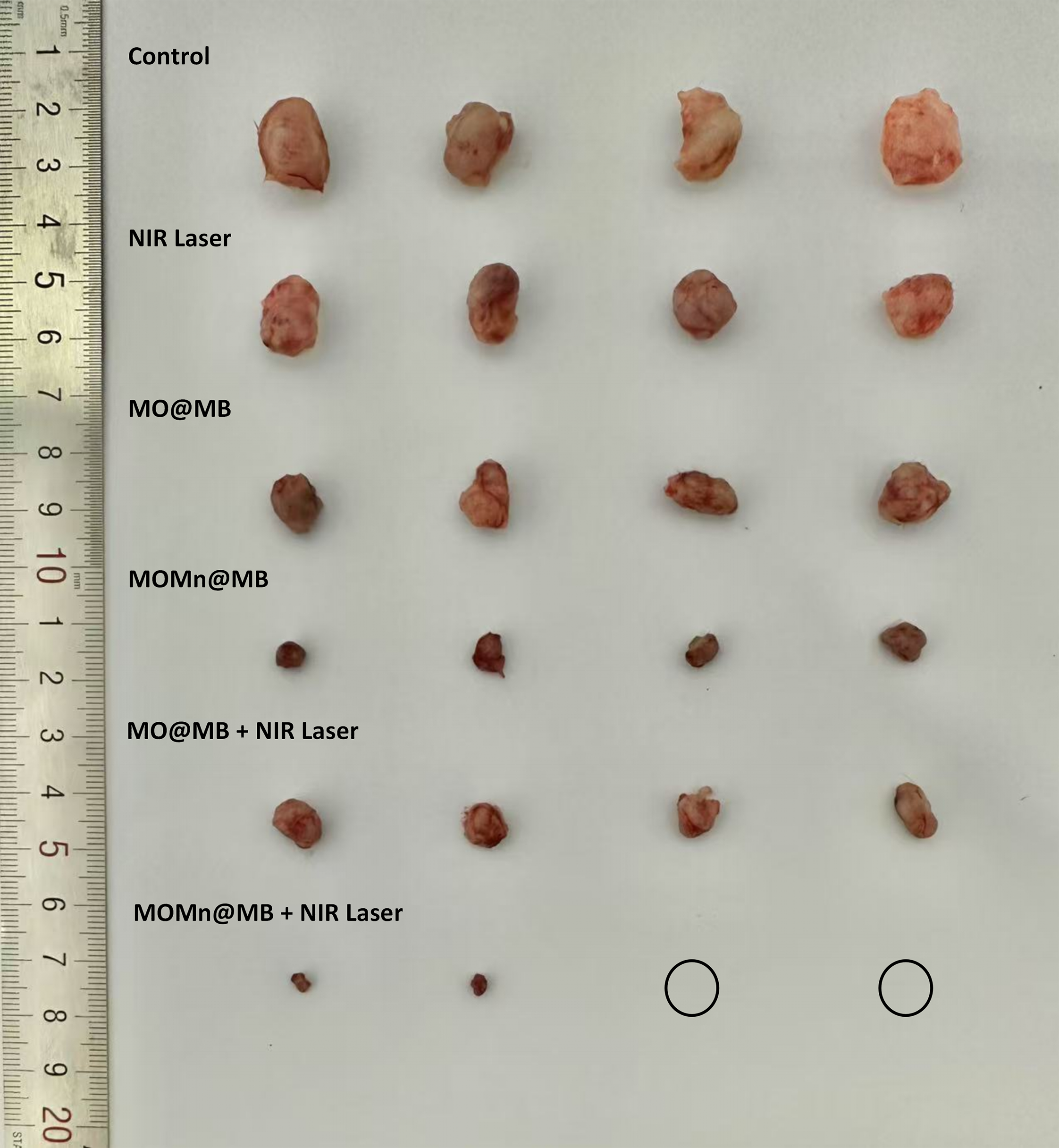


**Figure S22** Digital photographs of tumors from mice with different treatments.


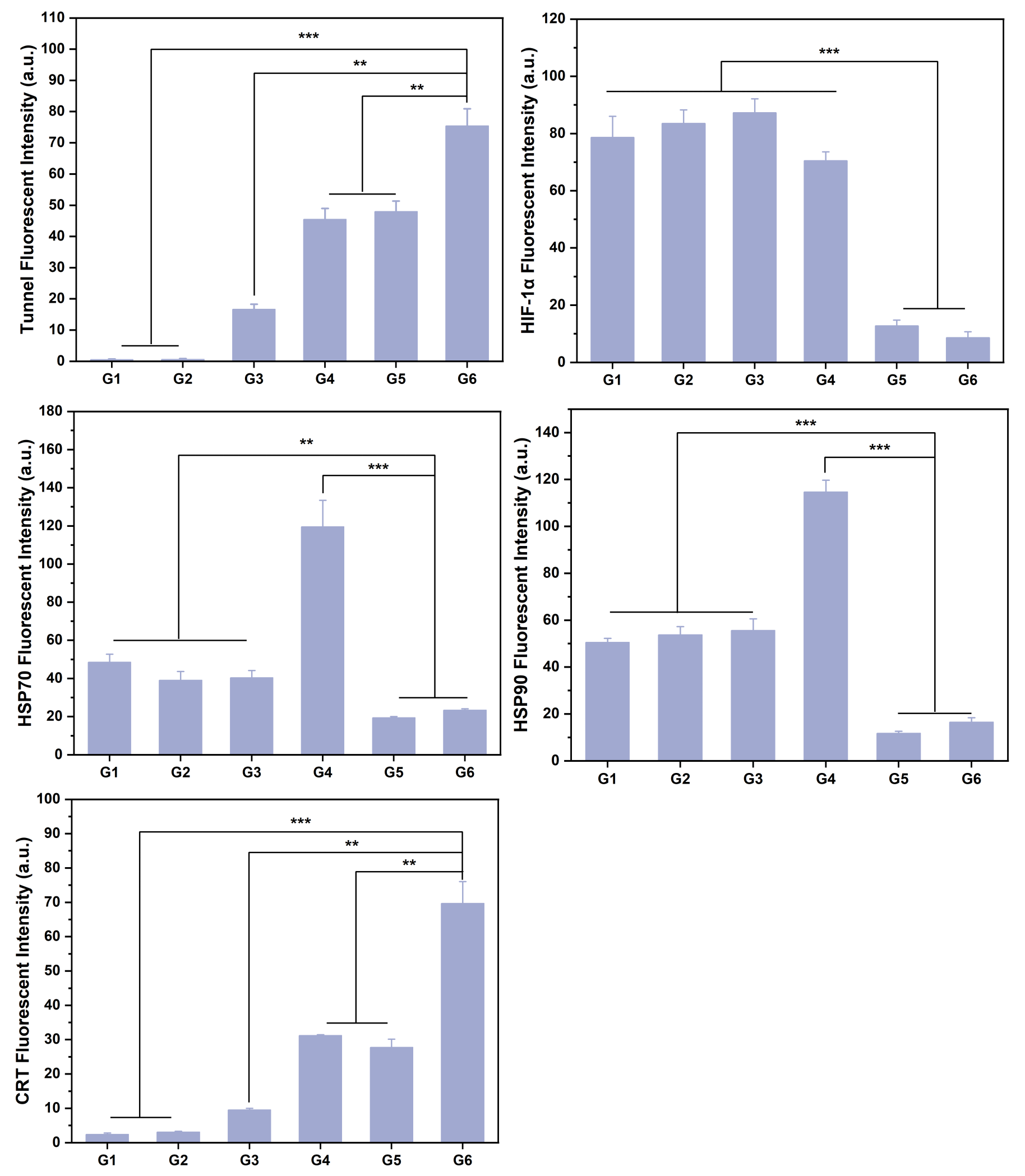


**Figure S23** Fluorescent intensity of Tunnel, HIF-1α, HSP70, HSP90 and CRT. **p* < 0.05, ***p* < 0.01, ****p* < 0.001.


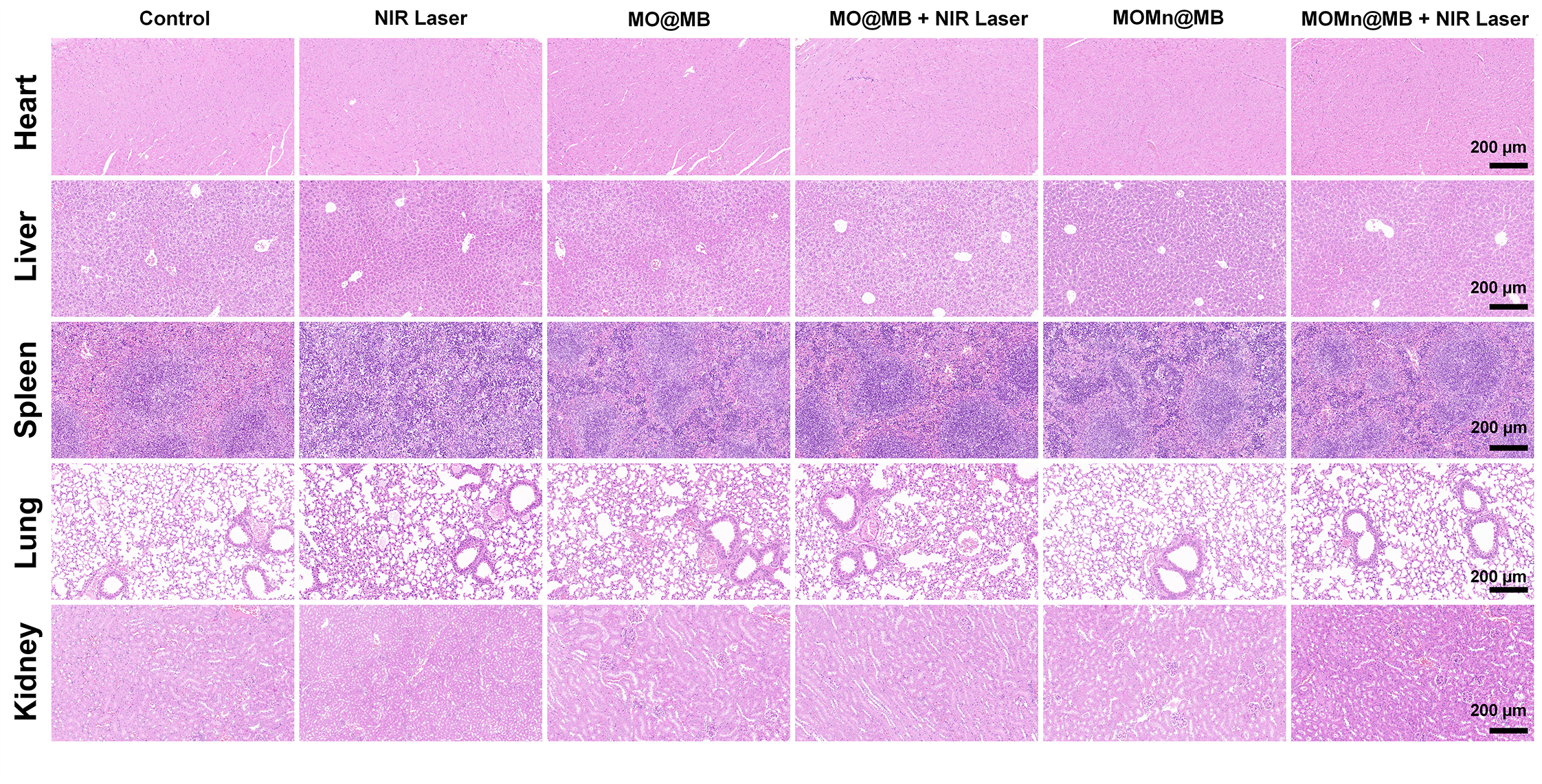


**Figure S24** H&E-stained sections of major organs of mice after different treatments.


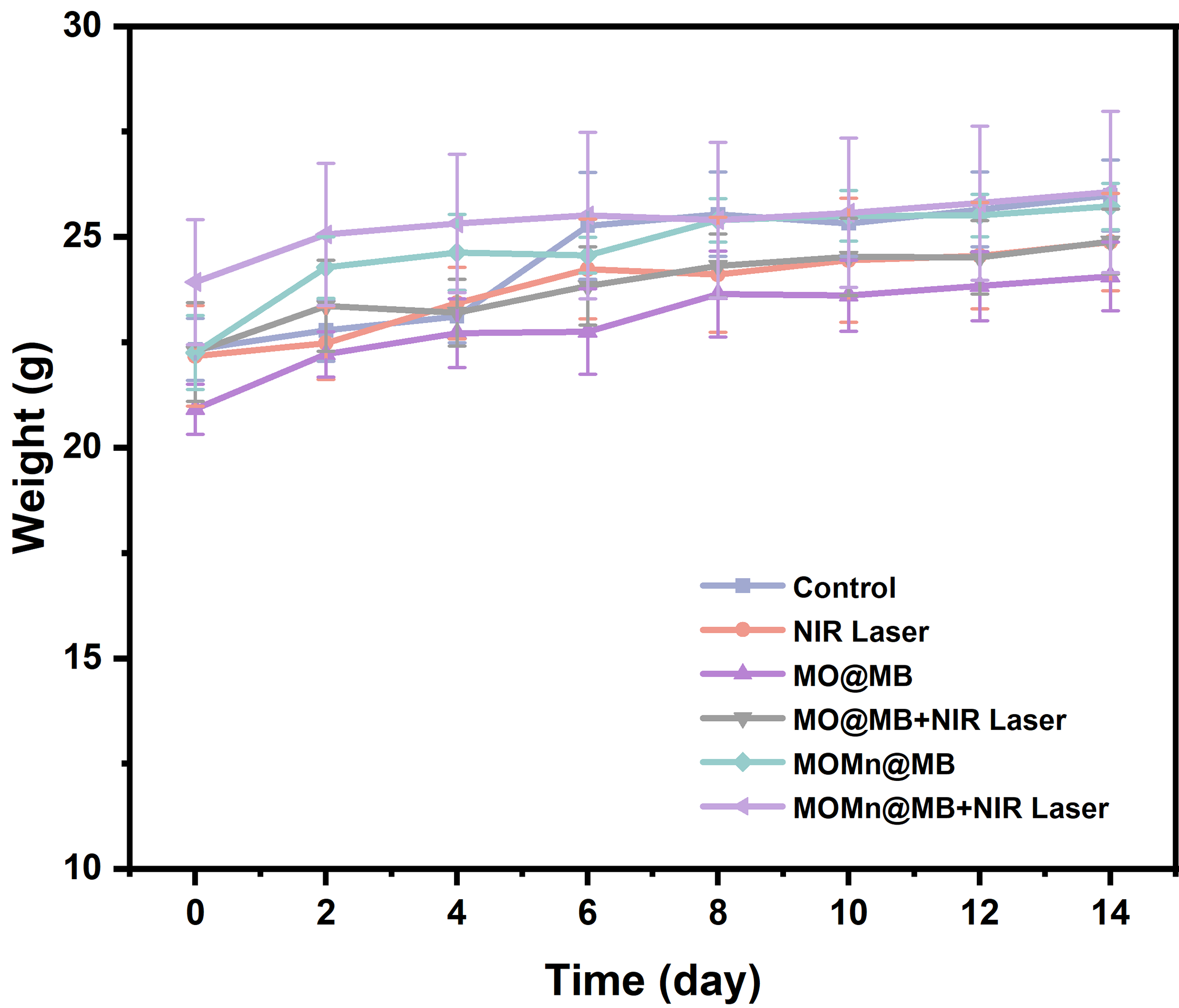


**Figure S25** Body weight curves of mice after different treatments.


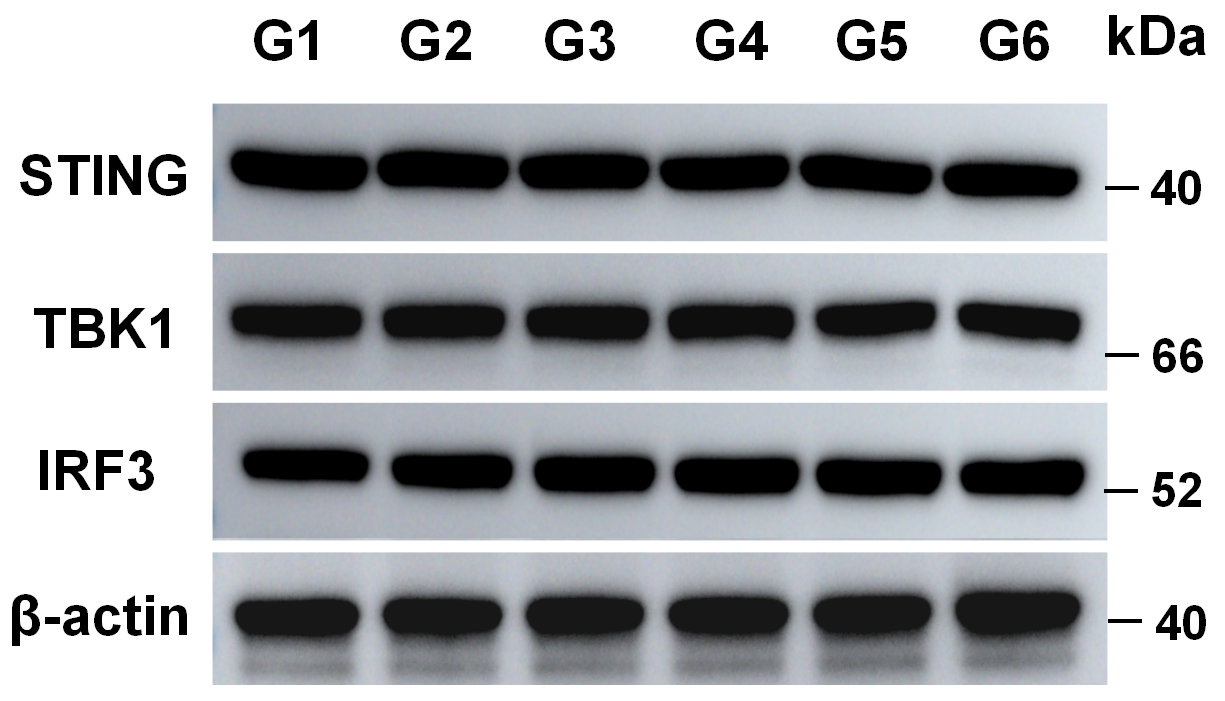


**Figure S26** Western blot analysis of cGAS-STING pathway-associated non-phosphorylated proteins in tumor tissues with different treatments.


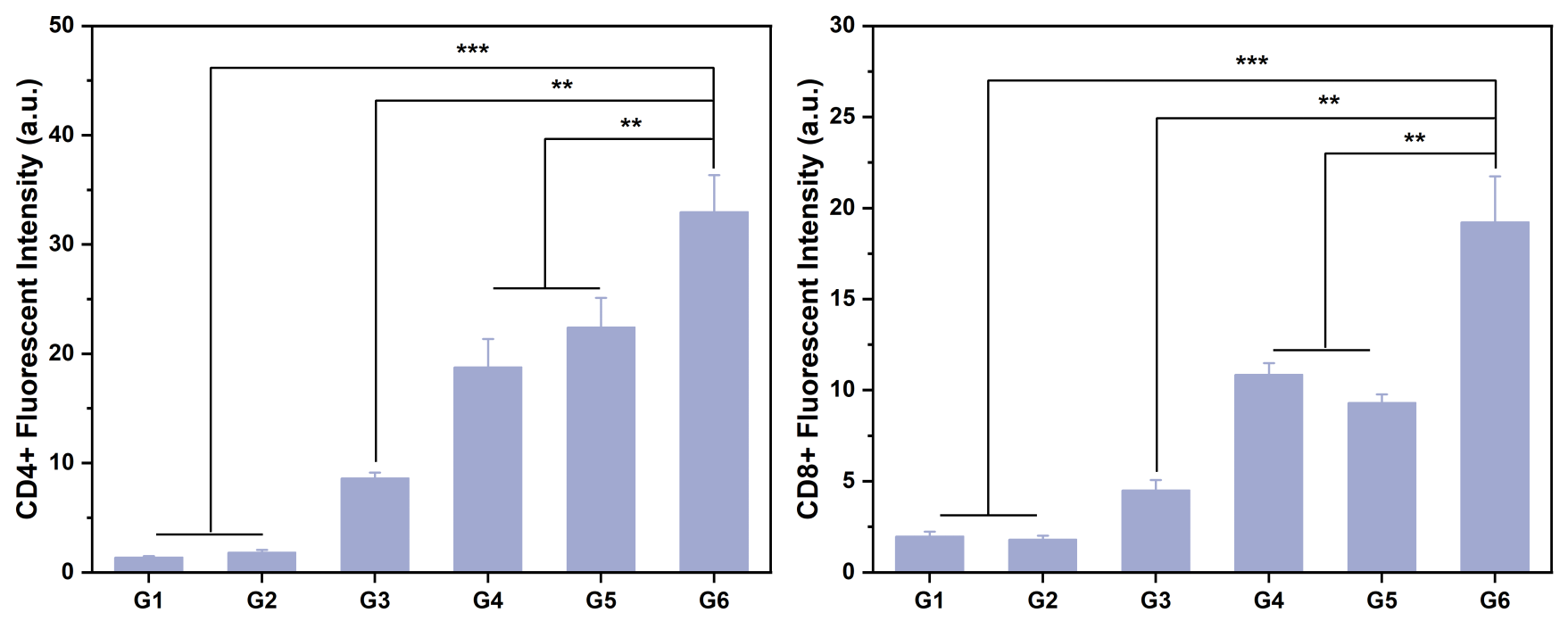


**Figure S27** Fluorescent intensity of CD4+ and CD8+ T cells. **p* < 0.05, ***p* < 0.01, ****p* < 0.001.


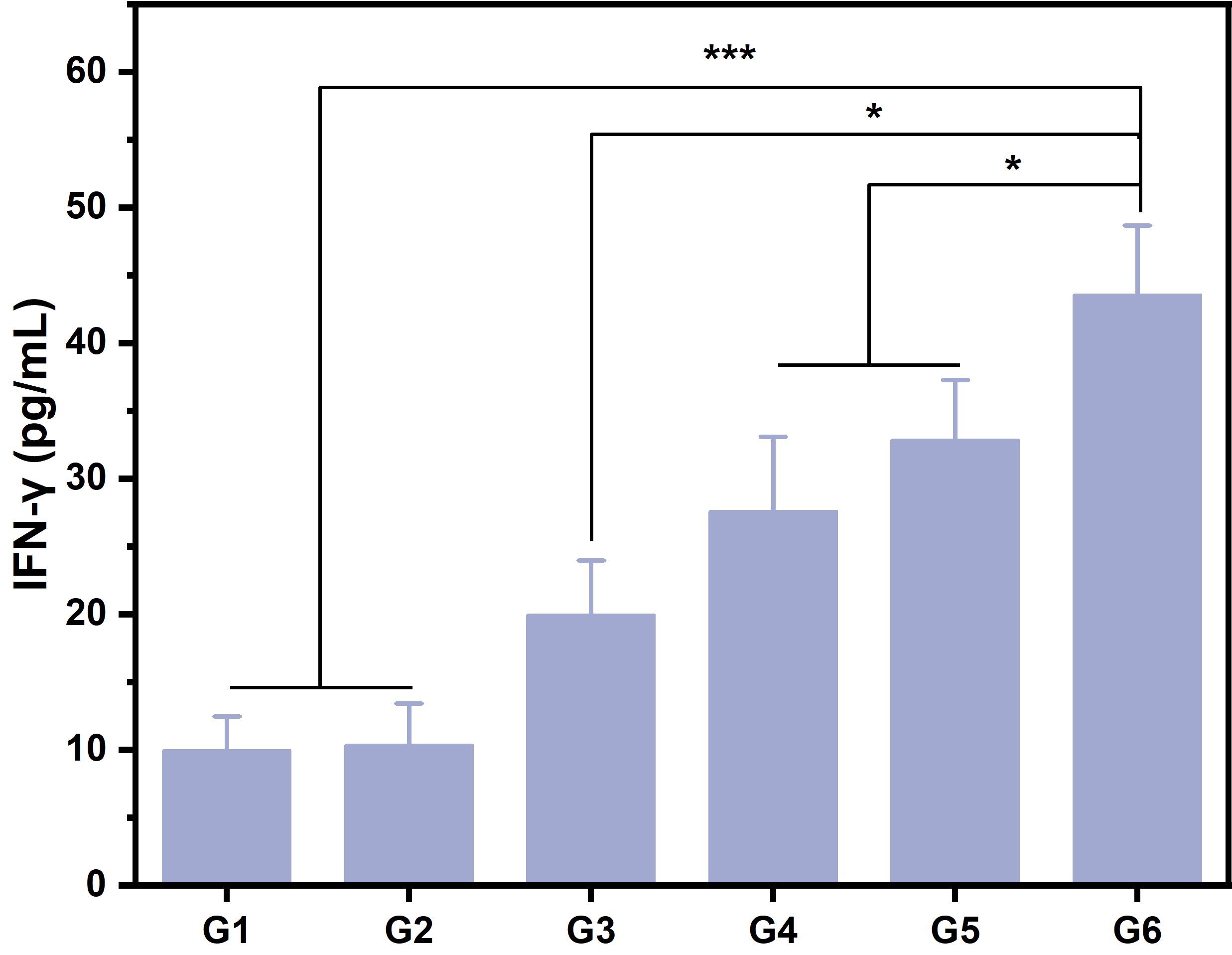


**Figure S28** Release of IFN-γ in serum of mice with different treatments. **p* < 0.05, ***p* < 0.01.


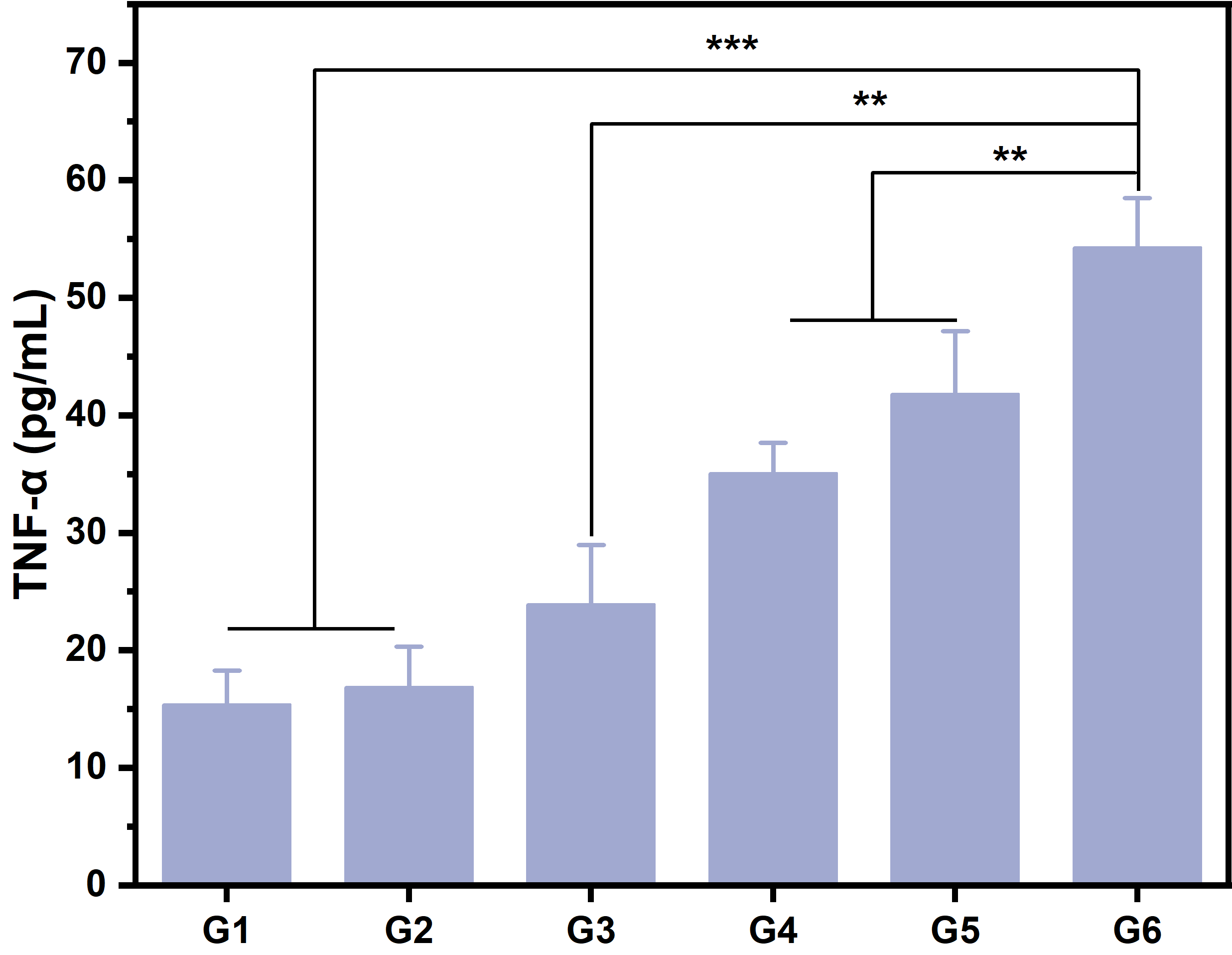


**Figure S29** Release of TNF-α in serum of mice with different treatments. **p* < 0.05, ***p* < 0.01, ****p* < 0.001.


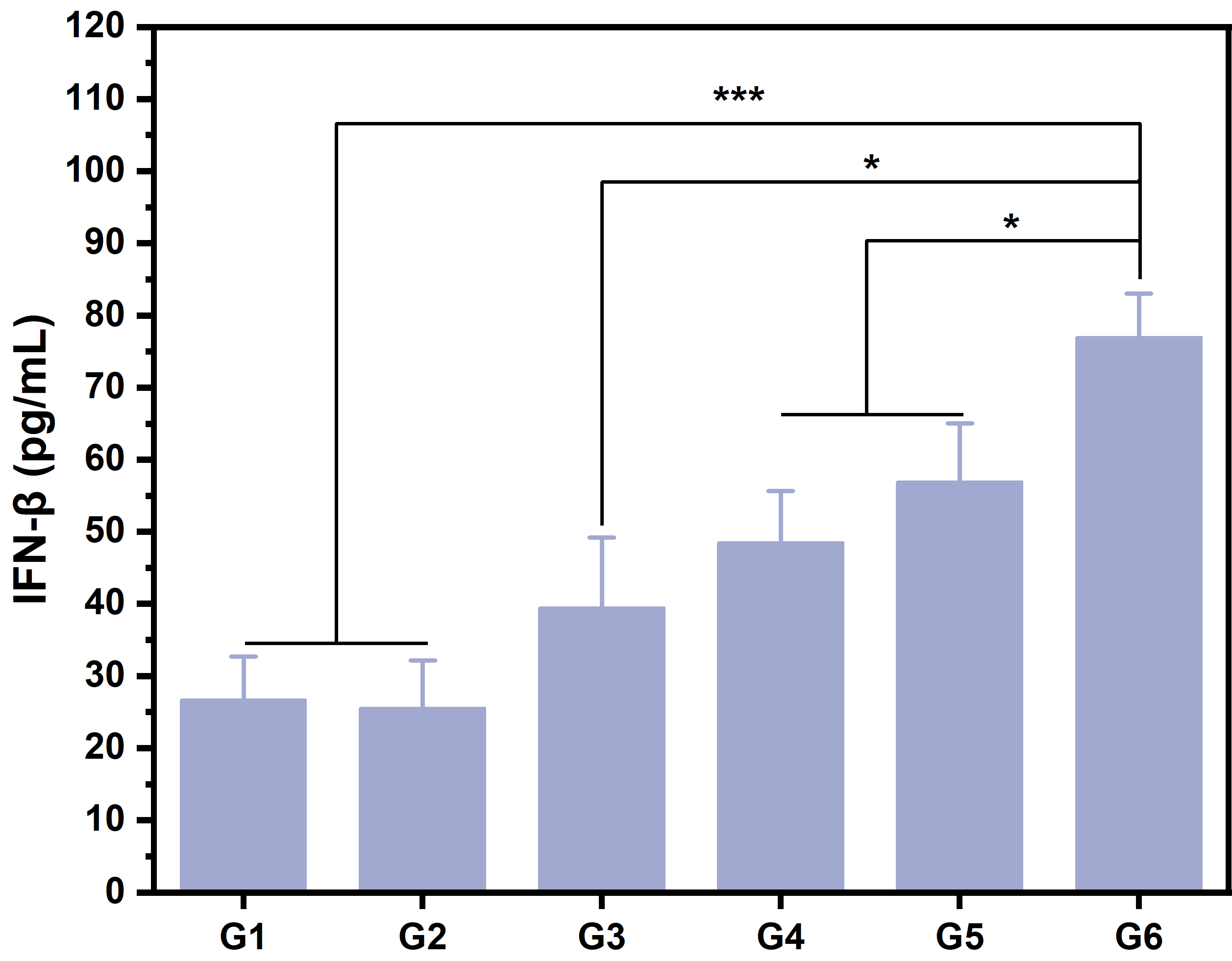


**Figure S30** Release of IFN-β in serum of mice with different treatments. **p* < 0.05, ***p* < 0.01.


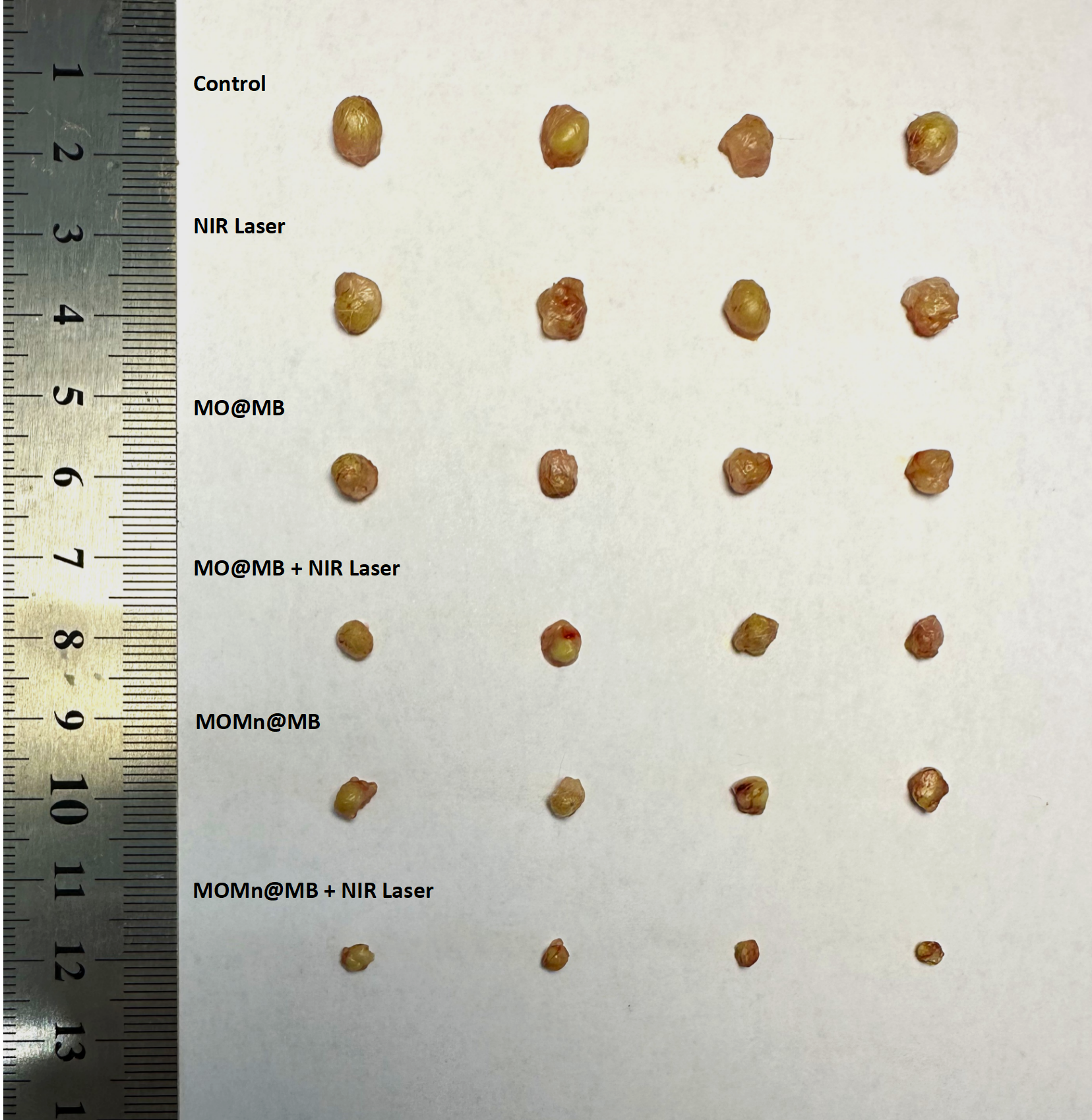


**Figure S31** Digital photographs of re-challenge tumors from mice with different treatments.
